# Genetic contributions to alcohol use disorder treatment outcomes: a genome-wide pharmacogenomics study

**DOI:** 10.1038/s41386-021-01097-0

**Published:** 2021-07-23

**Authors:** Joanna M. Biernacka, Brandon J. Coombes, Anthony Batzler, Ada Man-Choi Ho, Jennifer R. Geske, Josef Frank, Colin Hodgkinson, Michelle Skime, Colin Colby, Lea Zillich, Sofia Pozsonyiova, Ming-Fen Ho, Falk Kiefer, Marcella Rietschel, Richard Weinshilboum, Stephanie S. O’Malley, Karl Mann, Ray Anton, David Goldman, Victor M. Karpyak

**Affiliations:** 1 Department of Quantitative Health Sciences, Mayo Clinic, Rochester, MN USA; 2 Department of Psychiatry and Psychology, Mayo Clinic, Rochester, MN USA; 3 Department of Genetic Epidemiology in Psychiatry, Central Institute of Mental Health, Medical Faculty Mannheim, Heidelberg University, Mannheim, Germany; 4 National Institute on Alcohol Abuse and Alcoholism, Rockville, MD USA; 5 Department of Molecular Pharmacology and Experimental Therapeutics, Mayo Clinic, Rochester, MN USA; 6 Department of Addictive Behavior and Addiction Medicine, Central Institute of Mental Health, Medical Faculty Mannheim, Heidelberg University, Mannheim, Germany; 7 Yale School of Medicine, New Haven, CT USA; 8 Medical University of South Carolina, Charleston, SC USA

**Keywords:** Predictive markers, Behavioural genetics

## Abstract

Naltrexone can aid in reducing alcohol consumption, while acamprosate supports abstinence; however, not all patients with alcohol use disorder (AUD) benefit from these treatments. Here we present the first genome-wide association study of AUD treatment outcomes based on data from the COMBINE and PREDICT studies of acamprosate and naltrexone, and the Mayo Clinic CITA study of acamprosate. Primary analyses focused on treatment outcomes regardless of pharmacological intervention and were followed by drug-stratified analyses to identify treatment-specific pharmacogenomic predictors of acamprosate and naltrexone response. Treatment outcomes were defined as: (1) time until relapse to any drinking (TR) and (2) time until relapse to heavy drinking (THR; ≥ 5 drinks for men, ≥4 drinks for women in a day), during the first 3 months of treatment. Analyses were performed within each dataset, followed by meta-analysis across the studies (*N* = 1083 European ancestry participants). Single nucleotide polymorphisms (SNPs) in the *BRE* gene were associated with THR (min *p* = 1.6E−8) in the entire sample, while two intergenic SNPs were associated with medication-specific outcomes (naltrexone THR: rs12749274, *p* = 3.9E−8; acamprosate TR: rs77583603, *p* = 3.1E−9). The top association signal for TR (*p* = 7.7E−8) and second strongest signal in the THR (*p* = 6.1E−8) analysis of naltrexone-treated patients maps to *PTPRD*, a gene previously implicated in addiction phenotypes in human and animal studies. Leave-one-out polygenic risk score analyses showed significant associations with TR (*p* = 3.7E−4) and THR (*p* = 2.6E−4). This study provides the first evidence of a polygenic effect on AUD treatment response, and identifies genetic variants associated with potentially medication-specific effects on AUD treatment response.

## Introduction

Alcohol use disorder (AUD) is highly prevalent, and presents a significant health burden worldwide [1]. Several medications have been developed for treatment of AUD, but they are underutilized and pharmacological treatment of AUD remains a major challenge [2]. Recent reviews have highlighted the need for a precision medicine approach in the context of AUD treatment in order to increase the utility and safety of available medications [3, 4].

Treatment efficacy of acamprosate and naltrexone is supported by large systematic meta-analyses of randomized controlled trials [5, 6], which suggest that naltrexone helps people refrain from excessive drinking while acamprosate is effective in supporting abstinence [6–12]. However, a considerable proportion of AUD patients fail to benefit from these drugs, with the number needed to treat estimated to be 7–12 for acamprosate and 12–20 for naltrexone [7, 8, 13, 14]. Garbutt et al. reviewed moderators of naltrexone response and concluded that available data were insufficient to guide clinical treatment selection [15]. Similarly, limited success was achieved in studies searching for clinical moderators of acamprosate response [16]. Although secondary analyses have identified potential moderators of naltrexone and acamprosate response [17], further efforts are needed to identify predictors for personalized medicine [18].

Genetic variation contributes to inter-individual differences in drug response [19–22], and may facilitate the prediction of response to AUD treatment [23, 24]. Moreover, discovery of genetic variation and biological pathways involved in treatment response may reveal information about the mechanisms of drug action, accelerating further drug discovery efforts. Candidate gene studies identified several genetic variations that may influence AUD treatment outcomes [25–28]. However, the contribution of candidate gene studies to the understanding of genetic effects on AUD treatment response has been limited, highlighting the need for genome-wide pharmacogenomic studies. Yet, to date, no genome-wide association studies (GWASs) of acamprosate or naltrexone treatment response have been published.

Recent GWASs of AUD and other alcohol use related traits have identified a growing number of loci associated with these phenotypes. Various downstream analyses including gene, pathway and polygenic risk score (PRS) analyses have provided further insights into the genetic architecture of AUD and related traits, including genetic overlap with other psychiatric and behavioral traits [29–33]. While identifying loci contributing to AUD has been challenging due to the complexity of AUD and difficulties with compiling large samples of patients evaluated for this phenotype, GWAS of quantitative measures of problem drinking and alcohol consumption have been more successful [29–33]. Despite its clinical relevance, AUD treatment response has not yet been studied with the GWAS approach. Investigating the genetics of AUD treatment response is expected to not only reveal genetic variation potentially useful in treatment selection but may also contribute to our understanding of AUD risk and prognosis.

GWAS of treatment outcomes have been performed in the context of other addictions, most notably nicotine dependence. Large studies of smoking behavior phenotypes, including smoking cessation, demonstrated shared and unique genetic contributions across these phenotypes, nicotine use disorder, and other diseases [34–36]. Studies of nicotine clearance and nicotine metabolism biomarkers have provided further insights regarding the genetic contributions to complex smoking related phenotypes [37, 38]. However, similar to AUD, little is known about pharmacogenetic factors that contribute to response to specific smoking cessation treatments.

Here we present the first GWAS of AUD treatment response based on data from three of the largest studies of acamprosate and naltrexone completed to date, with a total sample of more than 1000 patients treated for AUD: the COMBINE [39], PREDICT [40], and CITA [27] studies. COMBINE and PREDICT were randomized, placebo-controlled studies designed to evaluate response to acamprosate and naltrexone, while CITA was an open-label study of acamprosate designed for pharmacogenomics analyses. Using data from these three studies, we performed GWAS of AUD treatment outcomes (across treatment options) as well as pharmacogenomics GWAS of acamprosate and naltrexone response, separately. Our analyses included gene-level tests, gene-set and tissue enrichment analyses, and PRS analyses. Our results provide the first evidence of a polygenic effect on AUD treatment outcomes.

## Methods

### Samples, genotyping, quality control, and imputation

This study used a new genomic dataset derived from three previously completed studies of acamprosate and/or naltrexone treatment of AUD: the COMBINE, PREDICT, and CITA studies [27, 39, 40]. Key characteristics of these three studies are summarized in the Supplementary text and Supplementary Table S1. All subjects included in our analyses provided consent allowing use of their clinical data and DNA for genetic studies of AUD and response to its treatment, and this study was approved by the Mayo Clinic Institutional Review Board.

Sample genotyping, quality control, and imputation are described in the Supplementary Methods. After quality control, data from 498 COMBINE participants, 266 PREDICT participants, and 319 CITA participants of European ancestry with treatment outcome data were available for analysis. Following imputation, 5.6 million SNPs with minor allele frequency (MAF) > 0.05 in at least one of the studies were included in the meta-analyses.

### Assessment of treatment outcomes

Detailed descriptions of study procedures and assessments in the COMBINE, PREDICT, and CITA studies are presented elsewhere [27, 39, 40]. In brief, baseline patient characteristics were collected in each study, and outcomes were assessed during 3 or more months of treatment. We compared baseline measures (demographic data and clinical information including alcohol consumption in the 30 days prior to treatment initiation) across studies using chi-square tests or one-way analysis of variance. Treatment outcomes were derived from timeline follow back (TLFB) data collected after treatment initiation. For this study, the primary treatment outcome measures were: (1) time until relapse (TR) to any drinking during the first 3 months of treatment, and (2) TR to heavy drinking (≥5 drinks for men, ≥4 drinks for women in a day) during the first 3 months of treatment. Outcomes for patients that were lost to follow-up in the first 3 months prior to relapse (or heavy relapse) were treated as censored observations in the survival analyses. Although the two outcomes we analyzed are highly correlated because for many patients first relapse was an episode of heavy drinking, we considered both outcomes, as acamprosate is believed to be effective in preventing relapse to any drinking while naltrexone has been reported to be more effective in preventing relapse to heavy drinking [6, 8, 9].

### Genome-wide association analyses

The primary analyses included patients treated with acamprosate, naltrexone, or placebo (*N* = 1083) to identify predictors of treatment outcomes regardless of pharmacological intervention. Drug-stratified analyses were then run to identify treatment-specific (i.e., pharmacogenomic) predictors of acamprosate (*N* = 652) and naltrexone response (*N* = 301). In these drug-stratified analyses, patients were included if they were treated with the medication of interest (acamprosate or naltrexone), irrespective of other co-therapies. Thus, the GWAS of naltrexone outcomes in the COMBINE dataset included patients regardless of whether they had received the combined behavioral intervention and whether they had received naltrexone co-therapy; this means the acamprosate and naltrexone samples overlapped by a subset of COMBINE subjects that had been treated with both medications (*N* = 101). All GWAS were run in COMBINE, PREDICT and CITA datasets separately, followed by fixed-effects meta-analysis. In each dataset, allelic associations with TR and time until heavy relapse (THR) were assessed using Cox proportional hazards models. All analyses were adjusted for genetic principal components (PCs), if needed, to control for remaining population stratification in the European ancestry samples. The Cox proportional hazards analyses were run using the survival package in R (version 3.6.2), with SNP effect sizes estimated using hazard ratios (HR). Meta-analyses of GWAS summary statistics were performed using METAL [41]. Methods for gene-level as well as gene-set and tissue enrichment analyses are described in the Supplementary Text.

### Polygenic risk score analyses

Leave-one-out PRS analyses were used to test for a reproducible polygenic predictor of treatment outcomes between datasets. Specifically, leave-one-out PRSs for TR and THR were generated for each target study based on the results of a discovery GWAS meta-analysis where the target cohort was left out. The PRSs were constructed using PRSice2 [42] to prune (*r*^2^ > 0.1 within a 500 kb window) and restrict SNPs to a given *p* value threshold (p_t_ = 0.0001, 0.001, 0.01, 0.05, 0.1, 0.2, 1), with SNP alleles weighted by their log(HR) estimates. We then performed a principal component analysis (PCA) on the set of PRSs estimated at different *p* value thresholds and used the first PRS principal component to test for association with the outcome; this PRS-PCA strategy eliminates the multiple testing across PRSs based on different *p* value thresholds [43]. The PRSs for TR and THR were tested for association with the respective treatment outcome in each left out dataset using Cox proportional hazards models, and the results from the analyses of the three datasets were meta-analyzed to assess overall PRS prediction of treatment response.

To investigate whether PRS for other AUD-related traits predict AUD treatment outcomes, we generated PRS for AUD and alcohol consumption using summary statistics from several published GWAS. For AUD, we derived the PRS based on the largest clinically assessed AUD sample [32], a multi-ethnic sample with ICD diagnoses of AUD [31], and a large meta-analysis of problematic alcohol use [33]. For alcohol consumption, we used PRS derived from the UK Biobank GWAS [29] and from the Million Veterans Program data [31]. These PRSs were also constructed using PRSice2 [42] (*r*^2^ > 0.1 within a 500 kb window; p_t_ = 5e−8, 1e−7, 1e−06, 1e−5, 0.0001, 0.001, 0.01, 0.05, 0.1, 0.2, 1). As with the leave-one-out analyses, we used the PRS-PCA strategy to reduce multiple testing across *p* value thresholds by computing a single PRS for each set of GWAS summary statistics and testing its association with the outcome.

The PRS analyses of the COMBINE study data were adjusted for the first four PCs to account for the heterogeneity observed in this dataset. Nagelkerke’s pseudo-*R*^2^ was calculated to estimate the variance explained in TR or THR by the PRS in each dataset, and weighted average *R*^2^ values were calculated across the three datasets using the effective N for each cohort.

## Results

Descriptive statistics for the three datasets are shown in Table 1. In total, 639 (59%) of the study participants relapsed to any drinking, while 564 (52%) relapsed to heavy drinking.

**Table 1:** Demographic and clinical characteristics of the COMBINE, PREDICT, and CITA participants included in the pharmacogenomics GWAS.

	COMBINE	PREDICT	CITA	
Sample sizes				*p* value
Total N	498	266	319	
Acamprosate N	223	110	319	
Naltrexone N	199	102	–	
Placebo only or no pills N	177	54	–	
Age, mean(SD)	45.4 (10.6)	45.2 (8.5)	43.4 (11.6)	0.012
Sex, male N(%)	343 (68.9%)	266 (100%)	204 (64.0%)	<0.0001
Age of onset	30.5 (11.9)	30.2 (10.2)	29.6 (12.0)	0.63
Baseline alcohol consumption^a^, mean (sd)				
Days since last drinking day	8.0 (5.4)	22.0 (4.3)	19.7 (8.8)	<0.0001
Average number of drinks per drinking day	12.1 (7.4)	21.0 (12.7)	12.0 (8.6)	<0.0001
% Drinking days^b^	56.2 (22.8)	82.0 (26.8)	32.2 (28.2)	<0.0001
% heavy drinking days	47.2 (23.8)	79.4 (27.9)	28.6 (26.8)	<0.0001
Treatment Outcomes:				
Relapse, N (%)	380 (76.3%)	158 (59.4%)	101 (31.7%)	<0.0001
Relapse in acamprosate subset, N (%)	167 (74.9%)	62 (56.4%)	101 (31.7%)	<0.0001
Relapse in naltrexone subset, N (%)	146 (73.4%)	65 (63.7%)	–	0.084
Heavy Relapse, N (%)	338 (67.9%)	142 (53.4%)	84 (26.3%)	<0.0001
Heavy relapse in acamprosate subset, N (%)	144 (64.6%)	56 (50.9%)	84 (26.3%)	<0.0001
Heavy relapse in naltrexone subset, N (%)	127 (63.8%)	57 (55.9%)	–	0.18

^a^Baseline alcohol consumption measures are based on 30 days before start of treatment.

^b^% drinking days = 100 − % days abstinent in the 30 days before start of treatment.

### Genome-wide association study

**Fig. 1:**
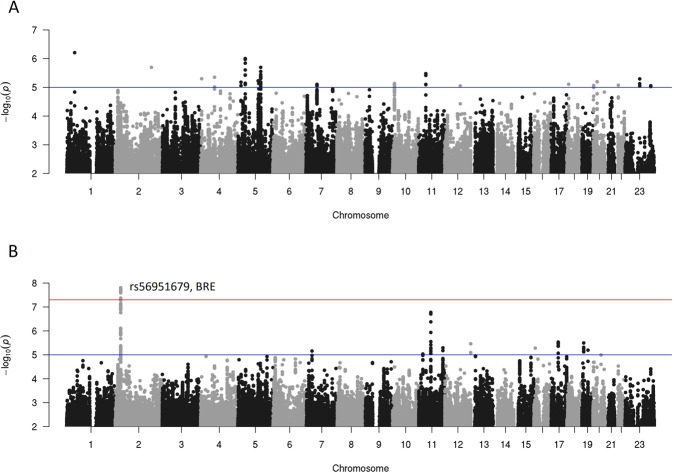
Manhattan plots for analyses of outcomes in all subjects. Manhattan plots are shown for (**A**) time until relapse to any drinking, and (**B**) time until relapse to heavy drinking. In each panel, -log10(pvalue)s are shown (y-axis) for all SNPs by SNP position in the genome (x-axis).

Manhattan plots for the two GWAS meta-analyses in the full cohort (all treatments) are shown in Fig. 1; the corresponding Quantile/Quantile plots are shown in Supplementary Fig. S1. In the meta-analysis across the three studies no individual SNPs were significantly associated with TR; the SNP with the strongest evidence for association is located in an intron of the potassium voltage-gated channel subfamily Q member 4 gene (*KCNQ4*, rs1078110, MAF = 0.30, *P* = 6.2E−7). In the full cohort analysis of THR, significant associations were observed for a set of SNPs in the brain and reproductive organ-expressed gene *BRE*, also known as BABAM2 (BRISC and BRCA1 A complex member 2); there were 14 genome-wide significant SNPs at this locus (top SNP rs56951679, MAF = 0.17, minor allele HR = 1.5, *p* = 1.6E−8). The effect estimate for the top SNP at this locus (rs56951679) suggests that carrying one additional copy of the minor allele (C) is associated with 1.5 times higher risk of relapse to heavy drinking. A regional association plot for this locus is shown in Supplementary Fig. S2.

In drug-stratified meta-analyses of outcomes in patients treated with naltrexone (Supplementary Fig. S3), an intergenic SNP located between the long intergenic non-coding RNA RP4-710M16.2 and the *PPAP2B* gene (phospholipid phosphatase 3, also known as PLPP3) was associated with THR (rs12749274, MAF = 0.079, HR = 2.90, *p* = 3.9E−8). While not achieving genome-wide significance, the strongest evidence for association with TR and second strongest for THR was observed with a SNP in an intron of the protein tyrosine phosphatase receptor type D gene *PTPRD* (rs62533259, MAF = 0.14: TR minor allele HR = 2.2, *p* = 7.7E−8; THR minor allele HR = 2.4, *p* = 6.1E−8). *PTPRD* has been implicated in multiple addiction-related phenotypes in humans and other species [44].

One SNP was significantly associated with TR during acamprosate treatment; this intergenic SNP is located between a non-coding RNA gene and the ribosomal protein L29 pseudogene (rs77583603, MAF = 0.093, *p* = 3.1E−9; Supplementary Fig. S4). The top signal in the analyses of THR in acamprosate-treated patients was rs34797278 in a non-coding RNA (MAF = 0.079, *p* = 5.4E−8). All loci with suggestive evidence of association (*p* < 5*10^−6^) with one of the outcome measures are shown in Supplementary Tables S1 (all treatments), S2 (naltrexone), and S3 (acamprosate).

The top association findings described above were identified using meta-analyses of COMBINE, PREDICT, and CITA results. As these studies had significant differences in patient populations, we examined Forest Plots for the top loci to investigate the consistency of observed associations across these heterogeneous samples (Supplementary Fig. S5). The results were highly consistent across samples at the genome-wide significant loci as shown in Supplementary Fig. S5A/C/D. However, the top *PTPRD* SNP association that almost reached significance in the meta-analysis of naltrexone-treated patients in COMBINE and PREDICT, was driven by a genome-wide significant association in the COMBINE sample, while showing no significant association in the PREDICT sample, although the direction of effect in PREDICT was consistent with the effect in COMBINE and the 95% confidence intervals overlapped (Supplementary Fig. S5B).

**Fig. 2:**
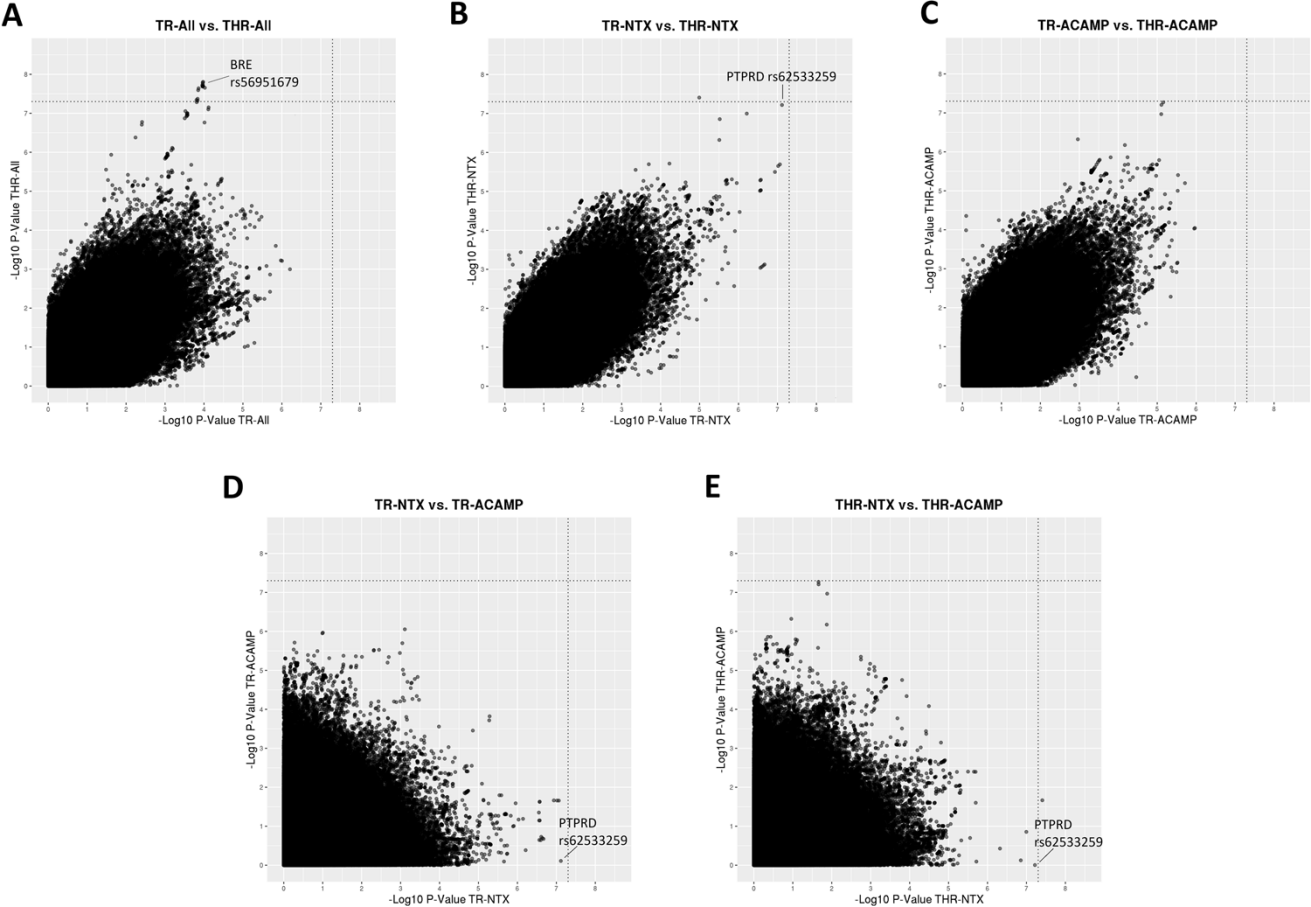
Scatterplots comparing results (*p* values) from GWAS of TR vs. THR and comparing results of analyses of different patient subsets. Scatterplots of -log(p) for (**A**) analysis of TR vs. analysis of THR in all subjects, (**B**) analysis of TR vs. analysis of THR in naltrexone-treated subjects, (**C**) analysis of TR vs. analysis of THR in acamprosate-treated subjects, (**D**) analysis of TR in acamprosate-treated patients vs. analysis of TR in naltrexone-treated patients, and (**E**) analysis of THR in acamprosate-treated patients vs. analysis of THR in naltrexone-treated patients.

Figure 2 shows scatterplots of results (*p* values) from analyses of TR vs. THR, and for analyses of acamprosate treatment outcomes vs. naltrexone treatment outcomes. As expected, TR GWAS signals were highly correlated with THR signals (panels A, B, and C of Fig. 2), but results for acamprosate treatment outcomes showed little correlation with signals for naltrexone treatment outcomes (panels D and E of Fig. 2). Although TR results were generally highly correlated with THR results, the top association from the THR analysis of the full cohort (the *BRE* top SNP *p* = 1.6E−8) showed much weaker evidence for association with TR (*p* = 1E−0). On the other hand, the *PTPRD* rs62533259 association in the naltrexone-treated subset was a top signal in both the TR and THR analyses (shown in panel B of Fig. 2). However, the same *PTPRD* variants were not associated with TR or THR in the acamprosate-treated patient subset (panels D and E of Fig. 2).

Results of the gene-level, gene-set, and tissue enrichment analyses are described in the Supplementary Text.

### Polygenic risk score analyses

**Fig. 3:**
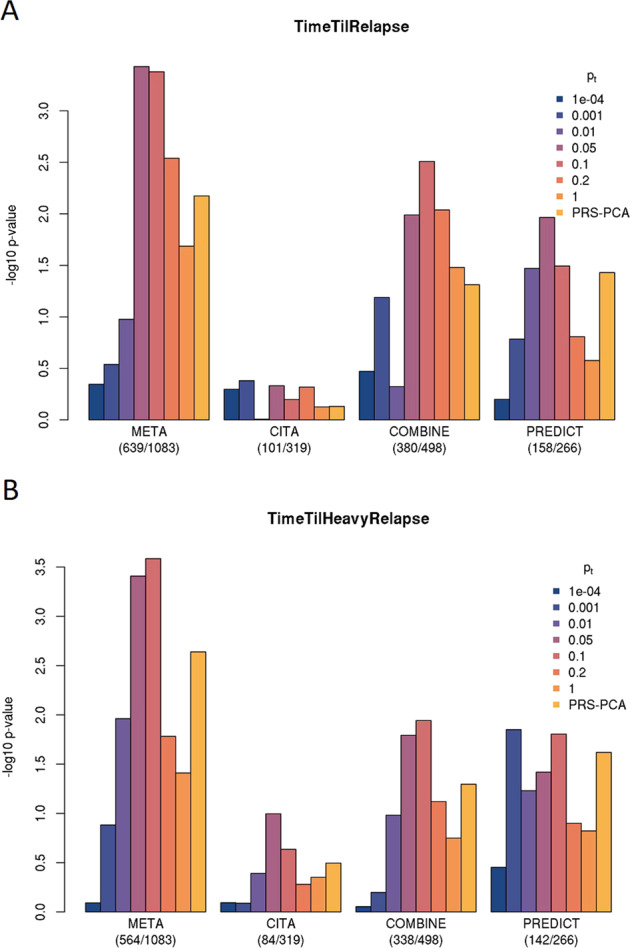
Leave-one-out PRS analyses. Leave-one-out PRS analysis of (**A**) time until relapse to any drinking and (**B**) time until relapse to heavy drinking. In each of the three studies (CITA, COMBINE and PREDICT) PRSs were constructed based on a discovery GWAS in the remaining two samples across a range of *p* value thresholds (p_T_ denoted using different colors described in the legend) to select SNPs for inclusion in the PRS. The selected SNPs (after LD pruning) were used to compute PRSs and the association of the PRSs with the outcome (TR or THR) was tested. The plots show the –log10(*p* values) for these association tests (on the *y*-axis) in each sample, as well as the meta-analysis of the leave-one-out PRS associations across the studies. The PRS association meta-analyses provided significant results for both time until relapse (*p* = 3.7E−04, Nagelkerke’s *R*^2^ = 1.3% at *p*_T_ = 0.05) and time until heavy relapse (*p* = 2.6E−04, Nagelkerke’s *R*^2^ = 1.3% at *p*_T_ = 0.10).

Leave-one-out analyses provided evidence of a polygenic effect on treatment response in AUD. Specifically, for both TR and THR, PRSs derived from SNP effects observed in any two of the studies explained a statistically significant proportion of variance in the same outcome in the left-out dataset (TR: Nagelkerke’s *R*^2^ = 0.8%, *p* = 0.007 with PRS-PCA, max *R*^2^ = 1.3%, *p* = 3.7E−4 at *p*_t_ = 0.05; THR: Nagelkerke’s *R*^2^ = 1.0%; *p* = 0.002 with PRS-PCA, max *R*^2^ = 1.3%, *p* = 2.6e−4 at *p*_t_ = 0.05; Fig. 3). In contrast, none of the PRSs for AUD, problematic alcohol use or alcohol consumption were associated with either treatment outcome (Supplementary Fig. S7).

## Discussion

Using data from three studies of AUD treatment outcomes, we performed the first GWAS for AUD treatment response, including drug-stratified pharmacogenomic analyses of acamprosate and naltrexone treatment outcomes, and identified several genome-wide significant associations. We also performed PRS analyses to assess if a polygenic signal captured by this GWAS is associated with AUD treatment outcomes in independent samples. Results of these leave-one-out PRS analyses provided the first evidence of association between AUD treatment outcomes and PRSs, reflecting combined effect of variation across the genome on response to AUD treatment. While the proportion of variation in the treatment response explained by the PRSs is small, as expected for relatively small studies of complex traits, the significant association demonstrates that polygenic effects contribute to AUD treatment outcomes motivating further research into identifying contributing genetic factors.

Significant evidence of association was observed between THR and SNPs in the brain and reproductive organ-expressed protein (*BRE*) gene (aliases include *BABAM2*, *BRCC4* and *BRCC45*), which encodes BRISC and BRACA1 A complex member 2. *BRE* is ubiquitously expressed in human tissues, but most prominently in the zona glomerulosa of the adrenal cortex, where mineralocorticoids (e.g., aldosterone) are synthesized and secreted, as well as in glia and neurons [45]. *BRE* expression is altered in adrenal abnormalities, suggesting possible involvement in adrenal function [45]. Chronic alcohol consumption has been shown to increase blood aldosterone levels, and increased aldosterone level was correlated with higher alcohol consumption, craving and anxiety levels in AUD patients [46]. Aldosterone may regulate alcohol use behaviors and anxiety via mineralocorticoid receptors expressed in limbic regions involved in regulating anxiety, stress-induced alcohol consumption, craving, and inhibitory control [47]. Thus, our finding of association of *BRE* SNPs with THR may be related to regulation of stress response and alcohol craving. Furthermore, *BRE* has shown genome-wide significant associations with a range of phenotypes from different domains (e.g., metabolic, immunological, cardiovascular) [48]. With respect to psychiatric traits, genetic variation in *BRE* has been associated with alcohol intake frequency and drinks per week in the general population [34]. In our study, *BRE* SNPs were significantly associated with THR, but they were not significantly associated with TR despite the overall correlation of results for these two outcomes. It is possible that this gene has a differential effect on relapse and heavy relapse (e.g., if it is involved in regulation of alcohol consumption quantity or AUD severity), particularly given the prior reports of *BRE* genetic variation being associated with alcohol intake frequency and drinks per week. However, it is also worth noting that according to GTEx, *BRE* rs56951679 (and other top SNPs in our analysis) is a splicing quantitative trait locus for *FNDC4* exon 7 in human cortex, hippocampus, and nucleus accumbens. FNDC4, a secreted factor highly expressed in liver and brain, was implicated in sex-specific neurotoxic consequences of chronic alcohol withdrawal [49]. The mechanisms of action through which variants in the *BRE* region may impact response to AUD treatment need to be further explored.

The one SNP with genome-wide significant evidence of association with acamprosate treatment response (rs77583603; *p* = 3.1E−9 for TR) is located between a non-coding RNA gene and the ribosomal protein L29 pseudogene and is not itself annotated as functionally significant. Analyses of naltrexone treatment outcomes identified a SNP near *PPAP2B* (a.k.a. *PLPP3, LPP3*), which encodes phospholipid phosphatase 3, that was significantly associated with THR. Changes in *PPAP2B* gene expression have been observed in nucleus accumbens and amygdala of rodents after voluntary ethanol consumption [50, 51], and *PPAP2B* has been implicated in alcoholic fatty liver in humans [52]. These genes/variants identified in the medication-specific analyses have not been previously implicated in AUD or other addiction-related traits in humans, and their potential role in AUD treatment response needs further investigation.

An intronic SNP of *PTPRD*, while not quite genome-wide significant, was the strongest association signal for TR (*p* = 7.7E−8) and the second strongest association for THR (*p* = 6.1E−8) in the naltrexone analysis. This finding is notable because *PTPRD* (protein tyrosine phosphatase receptor type D) has been implicated in multiple addiction-related phenotypes in humans and in animal studies [53, 54]. Prior studies have not provided genome-wide significant evidence for association of *PTPRD* alleles with any substance use disorders, but the *PTPRD* locus showed genome-wide significant associations in a GWAS of obsessive-compulsive traits [55], suggestive association in a GWAS of opioid cessation [56], and it appears to have pleiotropic effects across brain phenotypes [44]. Protein tyrosine phosphatases are signaling molecules that regulate a variety of cellular processes, and *PTPRD* likely plays a role in neuronal cell adhesion. Uhl and Martinez [44] provided a comprehensive review of *PTPRD* genetics and neurobiology, and discussed its potential role as a pharmacological target with effects on brain phenotypes. Of note, the *PTPRD* effect we observed appears to be a pharmacogenomic effect specific to naltrexone, as our study provided no evidence of association of the same *PTPRD* variant with acamprosate treatment response. The impact of *PTPRD* on naltrexone treatment outcomes is, therefore, intriguing and warrants further investigation.

We performed GWAS of treatment outcomes in the full cohort of patients from the COMBINE, PREDICT, and CITA studies, as well as drug-specific analyses of acamprosate and naltrexone treatment response. The drug-stratified analyses aim to identify genetic variants that impact response to a given drug. The analyses of the full sample may also identify pharmacogenomic effects, but have greater power to identify genetic variants that impact risk of relapse regardless of specific treatment—these variants may include markers of severity of AUD or AUD subtypes that confer differential risk of relapse following treatment. Two genes implicated in this study, *BRE* and *PTPRD*, were previously associated with AUD related phenotypes. Known AUD risk genes, particularly *ADH1B* [32], were not significant predictors of treatment response in our analyses. This may be partly due to a relatively small contribution of AUD risk genes to treatment response, which would be consistent with findings in other psychiatric disorders including major depressive disorder [57, 58]. In addition, low-frequency variants that are protective against AUD could have very low frequencies in our samples of AUD patients, reducing the power to detect their contribution to treatment response. Indeed, this may be the case with *ADH1B* variants that are protective against AUD and have low allele frequencies in populations of European ancestry. For example, the AUD-associated SNP rs1229984 in *ADH1B* has a frequency of ~5% in European populations [59]. In the COMBINE dataset this SNP had a frequency of only ~2%, whereas in CITA and PREDICT its frequency was ~1% and <1%, respectively. Because of its low frequency and poor imputation quality in the CITA and PREDICT samples, this SNP was not analyzed in these datasets. However, analysis of the COMBINE data provided nominal evidence of association for rs1229984 with TR (HR = 0.32, *p* = 0.016) and THR (HR = 0.27, *p* = 0.0053); the effect estimates suggest the minor allele at rs1229984, which is protective against AUD, may have a strong protective effect against relapse during treatment, reducing the risk of relapse by about 70%. Because its MAF was below 0.05 in all of the samples, this SNP was excluded from the meta-analyses. We also found that PRSs for AUD and alcohol consumption were not associated with treatment response outcomes. These results stand in contrast to the significant prediction of the leave-one-out PRS for both TR and THR, suggesting that the genetic mechanisms involved in treatment response are different from the genetic mechanisms affecting consumption in non-AUD samples and addiction to alcohol.

This study made use of data from three prior studies of AUD treatment. These three studies have important similarities allowing for combined analysis, including use of the TLFB to assess drinking outcomes. However, the three studies also differed in relevant patient characteristics. The available PREDICT study data were only from men, but the COMBINE and CITA study samples included 31% and 36% women, respectively. Reported pre-treatment baseline alcohol consumption also differed, with PREDICT participants having the highest (and CITA participants having the lowest) alcohol consumption at baseline and participants in the COMBINE study having the shortest duration of abstinence prior to treatment initiation. The studies also differed in rates of treatment outcomes, with the CITA sample having the lowest percentage of any-drinking relapse and heavy-drinking relapse. While this heterogeneity may have limited the replicability of findings between the three datasets and reduced power of the meta-analysis, the differences between the studies also mean that identified genetic associations are likely robust genetic predictors of treatment outcomes that apply to a broad range of patients. In fact, the genome-wide significant loci in our study showed highly consistent results across the different samples. The *PTPRD* signal was driven by a genome-wide significant association in the COMBINE sample, while showing no significant association in the PREDICT sample; however, the direction of effect was the same and the confidence intervals for the SNP effect in the two samples overlapped. A more in-depth analysis of potential effect modifiers or confounders of the observed *PTPRD* association in a larger sample of patients treated with naltrexone is warranted.

In addition to sample heterogeneity that may have reduced the power of this study, other limitations need to be noted. The sample size of this study is smaller than what is typically required for well-powered GWAS of complex traits, particularly when the goal is identification of specific genetic risk variants. With a genome-wide significance threshold of 5e−8 and our sample of *N* = 1083 subjects, we had 80% power to detect SNP effects of HR = 1.80 and HR = 1.87 for TR and THR, respectively, for variants with MAF = 0.1. For more common variants with MAF = 0.4, our sample provided 80% power to detect SNP effects of 1.43 and 1.47 for the two outcomes, respectively. While genome-wide significant associations with several SNPs were identified in the full cohort and in the drug-stratified analyses, no significant gene-level associations were detected by MAGMA analysis. In the GWAS we used the standard significance threshold of 5e−8, although several different GWAS were performed (two outcomes, in the full sample as well as in drug-stratified samples). A Bonferroni correction for the two treatment outcome measures would not be appropriate as these outcomes are highly correlated, but the exploratory analyses of naltrexone and acamprosate treatment outcomes were largely independent. Thus, the observed SNP-level associations in these analyses should be interpreted cautiously and need to be replicated in independent samples. We also note that the sample size in this study was expected to be adequate to detect polygenic effects, and indeed our leave-one-out PRS results revealed a significant polygenic effect for both TR and THR.

Other limitations of this study include a lack of thorough investigation of the role of intermediate or potentially confounding factors such as demographic and clinical variables or comorbidities (e.g., smoking) as well as measures of AUD severity or alcohol consumptions. Because each of the contributing studies collected different data at baseline, harmonization of these variables and investigation of their role in the context of pharmacogenomics effects on AUD treatment response was beyond the scope of this study, but should be further investigated in the future. Similarly, the different study designs and limited sample size prohibited a formal evaluation of the specificity of findings in the drug-stratified analyses with SNP-drug interaction tests, which should be investigated in future studies. Finally, because of the limited ancestral diversity in the contributing studies, the analysis was limited to patients of European ancestry, limiting the generalizability of the findings to other populations, and reducing the power to identify effects of variants that are rare in European ancestry populations such as the *ADH1B* variant discussed above, or the *ALDH2* Glu504Lys polymorphism (rs671) that shows extraordinarily significant association to AUD, but only in populations where the locus is actually polymorphic. Studies of the genetics of AUD treatment response in samples with other ancestries are needed.

In conclusion, these first GWAS analyses of AUD treatment outcomes had limited power for discovery of specific genetic variants associated with response to AUD treatment. Nevertheless, they provided important insights, including the first demonstration of polygenic effects on AUD treatment outcomes, and identification of genetic variants potentially associated with AUD treatment response. These findings motivate further investigation of the genetic contribution to AUD outcomes and study of the biological mechanisms underlying response to medications used in the treatment of AUD.

## Supplementary information

Supplemental Material

## References

[1] GBD 2016 Alcohol and Drug Use Collaborators. (2018). The global burden of disease attributable to alcohol and drug use in 195 countries and territories, 1990-2016: a systematic analysis for the Global Burden of Disease Study 2016. Lancet Psychiatry.

[2] Litten RZ, Falk DE, Ryan ML, Fertig JB (2016). Discovery, Development, and Adoption of Medications to Treat Alcohol Use Disorder: goals for the Phases of Medications Development. Alcohol Clin Exp Res.

[3] Hartwell EE, Kranzler HR (2019). Pharmacogenetics of alcohol use disorder treatments: an update. Expert Opin Drug Metab Toxicol.

[4] Helton SG, Lohoff FW (2015). Pharmacogenetics of alcohol use disorders and comorbid psychiatric disorders. Psychiatry Res.

[5] Donoghue K, Elzerbi C, Saunders R, Whittington C, Pilling S, Drummond C, et al., The efficacy of acamprosate and naltrexone in the treatment of alcohol dependence, Europe versus the Rest of the World: a meta-analysis. Addiction. 2015;110:920–30. 10.1111/add.12875 25664494

[6] Jonas DE, Amick HR, Feltner C, Bobashev G, Thomas K, Wines R (2014). Pharmacotherapy for adults with alcohol use disorders in outpatient settings: a systematic review and meta-analysis. JAMA: J Am Med Assoc.

[7] Bouza C, Angeles M, Muñoz A, Amate JM (2004). Efficacy and safety of naltrexone and acamprosate in the treatment of alcohol dependence: a systematic review. Addiction.

[8] Rösner S, Leucht S, Lehert P, Soyka M (2008). Acamprosate supports abstinence, naltrexone prevents excessive drinking: evidence from a meta-analysis with unreported outcomes. J Psychopharmacol.

[9] Maisel NC, Blodgett JC, Wilbourne PL, Humphreys K, Finney JW (2013). Meta-analysis of naltrexone and acamprosate for treating alcohol use disorders: when are these medications most helpful?. Addiction.

[10] Dawson DA, Goldstein RB, Grant BF (2007). Rates and correlates of relapse among individuals in remission from DSM-IV alcohol dependence: a 3-year follow-up. Alcohol Clin Exp Res.

[11] Witkiewitz K, Saville K, Hamreus K (2012). Acamprosate for treatment of alcohol dependence: mechanisms, efficacy, and clinical utility. Ther Clin risk Manag.

[12] Cheng HY, McGuinness LA, Elbers RG, MacArthur GJ, Taylor A, McAleenan A (2020). Treatment interventions to maintain abstinence from alcohol in primary care: systematic review and network meta-analysis. BMJ.

[13] Rosner S, Hackl-Herrwerth A, Leucht S, Lehert P, Vecchi S, Soyka M, et al., Acamprosate for alcohol dependence. Cochrane Database Syst Rev. 2010:9:CD004332. 10.1002/14651858.CD004332.pub2 PMC12147086 20824837

[14] Heilig M, Egli M (2006). Pharmacological treatment of alcohol dependence: target symptoms and target mechanisms. Pharmacol Ther.

[15] Garbutt JC, Greenblatt AM, West SL, Morgan LC, Kampov-Polevoy A, Jordan HS (2014). Clinical and biological moderators of response to naltrexone in alcohol dependence: a systematic review of the evidence. Addiction.

[16] Verheul R, Lehert P, Geerlings PJ, Koeter MW, van den Brink W (2005). Predictors of acamprosate efficacy: results from a pooled analysis of seven European trials including 1485 alcohol-dependent patients. Psychopharmacology.

[17] Gueorguieva R, Wu R, O'Connor PG, Weisner C, Fucito LM, Hoffmann S (2014). Predictors of abstinence from heavy drinking during treatment in COMBINE and external validation in PREDICT. Alcohol Clin Exp Res.

[18] Litten RZ, Falk DE, Ryan ML, Fertig J, Leggio L (2020). Five Priority Areas for Improving Medications Development for Alcohol Use Disorder and Promoting Their Routine Use in Clinical Practice. Alcohol Clin Exp Res.

[19] Wilson JF, Weale ME, Smith AC, Gratrix F, Fletcher B, Thomas MG (2001). Population genetic structure of variable drug response. Nat Genet.

[20] Weinshilboum RM, Wang L (2006). Pharmacogenetics and pharmacogenomics: development, science, and translation. Annu Rev Genom Hum Genet.

[21] Wong ML, Dong C, Andreev V, Arcos-Burgos M, Licinio J (2012). Prediction of susceptibility to major depression by a model of interactions of multiple functional genetic variants and environmental factors. Mol Psychiatry.

[22] van der Wouden CH, van Rhenen MH, Jama W, Ingelman-Sundberg M, Lauschke VM, Konta L (2019). Development of the PGx-Passport: a Panel of Actionable Germline Genetic Variants for Pre-Emptive Pharmacogenetic Testing. Clin Pharm Ther.

[23] Kranzler HR, Edenberg HJ (2010). Pharmacogenetics of alcohol and alcohol dependence treatment. Curr Pharm Des.

[24] Litten RZ, Bradley AM, Moss HB (2010). Alcohol biomarkers in applied settings: recent advances and future research opportunities. Alcohol Clin Exp Res.

[25] Anton RF, Oroszi G, O'Malley S, Couper D, Swift R, Pettinati H (2008). An evaluation of mu-opioid receptor (OPRM1) as a predictor of naltrexone response in the treatment of alcohol dependence: results from the Combined Pharmacotherapies and Behavioral Interventions for Alcohol Dependence (COMBINE) study. Arch Gen Psychiatry.

[26] Kiefer F, Witt SH, Frank J, Richter A, Treutlein J, Lemenager T (2011). Involvement of the atrial natriuretic peptide transcription factor GATA4 in alcohol dependence, relapse risk and treatment response to acamprosate. Pharmacogenom J.

[27] Karpyak VM, Biernacka JM, Geske JR, Jenkins GD, Cunningham JM, Rüegg J (2014). Genetic markers associated with abstinence length in alcohol-dependent subjects treated with acamprosate. Transl Psychiatry.

[28] Spanagel R, Pendyala G, Abarca C, Zghoul T, Sanchis-Segura C, Magnone MC (2005). The clock gene Per2 influences the glutamatergic system and modulates alcohol consumption. Nat Med.

[29] Clarke TK, Adams MJ, Davies G, Howard DM, Hall LS, Padmanabhan S (2017). Genome-wide association study of alcohol consumption and genetic overlap with other health-related traits in UK Biobank (N=112 117). Mol Psychiatry.

[30] Deak JD, Miller AP, Gizer IR (2019). Genetics of alcohol use disorder: a review. Curr Opin Psychol.

[31] Kranzler HR, Zhou H, Kember RL, Vickers Smith R, Justice AC, Damrauer S (2019). Genome-wide association study of alcohol consumption and use disorder in 274,424 individuals from multiple populations. Nat Commun.

[32] Walters RK, Polimanti R, Johnson EC, McClintick JN, Adams MJ, Adkins AE (2018). Transancestral GWAS of alcohol dependence reveals common genetic underpinnings with psychiatric disorders. Nat Neurosci.

[33] Zhou H, Sealock JM, Sanchez-Roige S, Clarke TK, Levey DF, Cheng Z (2020). Genome-wide meta-analysis of problematic alcohol use in 435,563 individuals yields insights into biology and relationships with other traits. Nat Neurosci.

[34] Liu M, Jiang Y, Wedow R, Li Y, Brazel DM, Chen F (2019). Association studies of up to 1.2 million individuals yield new insights into the genetic etiology of tobacco and alcohol use. Nat Genet.

[35] Matoba N, Akiyama M, Ishigaki K, Kanai M, Takahashi A, Momozawa Y (2019). GWAS of smoking behaviour in 165,436 Japanese people reveals seven new loci and shared genetic architecture. Nat Hum Behav.

[36] Saccone NL, Emery LS, Sofer T, Gogarten SM, Becker DM, Bottinger EP (2018). Genome-Wide Association Study of Heavy Smoking and Daily/Nondaily Smoking in the Hispanic Community Health Study/Study of Latinos (HCHS/SOL). Nicotine Tob Res.

[37] Buchwald J, Chenoweth MJ, Palviainen T, Zhu G, Benner C, Gordon S, et al., Genome-wide association meta-analysis of nicotine metabolism and cigarette consumption measures in smokers of European descent. Mol Psychiatry. 2020. 10.1038/s41380-020-0702-z PMC7483250 32157176

[38] Chenoweth MJ, Ware JJ, Zhu A, Cole CB, Cox LS, Nollen N (2018). Genome-wide association study of a nicotine metabolism biomarker in African American smokers: impact of chromosome 19 genetic influences. Addiction.

[39] Anton RF, O'Malley SS, Ciraulo DA, Cisler RA, Couper D, Donovan DM (2006). Combined pharmacotherapies and behavioral interventions for alcohol dependence: the COMBINE study: a randomized controlled trial. JAMA.

[40] Mann K, Kiefer F, Smolka M, Gann H, Wellek S, Heinz A (2009). Searching for responders to acamprosate and naltrexone in alcoholism treatment: rationale and design of the PREDICT study. Alcohol Clin Exp Res.

[41] Willer CJ, Li Y, Abecasis GR (2010). METAL: fast and efficient meta-analysis of genomewide association scans. Bioinformatics.

[42] Choi SW, O’Reilly PF. PRSice-2: polygenic risk score software for biobank-scale data. Gigascience. 2019;8:giz082. 10.1093/gigascience/giz082 PMC6629542 31307061

[43] Coombes BJ, Ploner A, Bergen SE, Biernacka JM (2020). A principal component approach to improve association testing with polygenic risk scores. Genet Epidemiol.

[44] Uhl GR, Martinez MJ (2019). PTPRD: neurobiology, genetics, and initial pharmacology of a pleiotropic contributor to brain phenotypes. Ann N Y Acad Sci.

[45] Miao J, Panesar NS, Chan KT, Lai FM, Xia N, Wang Y (2001). Differential expression of a stress-modulating gene, BRE, in the adrenal gland, in adrenal neoplasia, and in abnormal adrenal tissues. J Histochem Cytochem.

[46] Aoun EG, Jimenez VA, Vendruscolo LF, Walter N, Barbier E, Ferrulli A (2018). A relationship between the aldosterone-mineralocorticoid receptor pathway and alcohol drinking: preliminary translational findings across rats, monkeys and humans. Mol Psychiatry.

[47] Koning A, Buurstede JC, van Weert L, Meijer OC (2019). Glucocorticoid and Mineralocorticoid Receptors in the Brain: a Transcriptional Perspective. J Endocr Soc.

[48] Watanabe K, Stringer S, Frei O, Umićević Mirkov M, de Leeuw C, Polderman T (2019). A global overview of pleiotropy and genetic architecture in complex traits. Nat Genet.

[49] Hashimoto JG, Wiren KM (2008). Neurotoxic consequences of chronic alcohol withdrawal: expression profiling reveals importance of gender over withdrawal severity. Neuropsychopharmacology.

[50] Lesscher HM, Houthuijzen JM, Groot Koerkamp MJ, Holstege FC, Vanderschuren LJ (2012). Amygdala 14-3-3ζ as a novel modulator of escalating alcohol intake in mice. PLoS ONE.

[51] Bell RL, Kimpel MW, McClintick JN, Strother WN, Carr LG, Liang T (2009). Gene expression changes in the nucleus accumbens of alcohol-preferring rats following chronic ethanol consumption. Pharm Biochem Behav.

[52] Covarrubias MY, Khan RL, Vadigepalli R, Hoek JB, Schwaber JS (2005). Chronic alcohol exposure alters transcription broadly in a key integrative brain nucleus for homeostasis: the nucleus tractus solitarius. Physiol Genom.

[53] Drgonova J, Walther D, Wang KJ, Hartstein GL, Lochte B, Troncoso J (2015). Mouse Model for Protein Tyrosine Phosphatase D (PTPRD) Associations with Restless Leg Syndrome or Willis-Ekbom Disease and Addiction: reduced Expression Alters Locomotion, Sleep Behaviors and Cocaine-Conditioned Place Preference. Mol Med.

[54] Uhl GR, Martinez MJ, Paik P, Sulima A, Bi GH, Iyer MR (2018). Cocaine reward is reduced by decreased expression of receptor-type protein tyrosine phosphatase D (PTPRD) and by a novel PTPRD antagonist. Proc Natl Acad Sci USA.

[55] Burton CL, Lemire M, Xiao B, Corfield EC, Erdman L, Bralten J (2021). Genome-wide association study of pediatric obsessive-compulsive traits: shared genetic risk between traits and disorder. Transl Psychiatry.

[56] Cox JW, Sherva RM, Lunetta KL, Johnson EC, Martin NG, Degenhardt L, et al., Genome-Wide Association Study of Opioid Cessation. J Clin Med. 2020;9:180. 10.3390/jcm9010180 PMC7019731 31936517

[57] García-González J, Tansey KE, Hauser J, Henigsberg N, Maier W, Mors O (2017). Pharmacogenetics of antidepressant response: a polygenic approach. Prog Neuropsychopharmacol Biol Psychiatry.

[58] Ward J, Graham N, Strawbridge RJ, Ferguson A, Jenkins G, Chen W (2018). Polygenic risk scores for major depressive disorder and neuroticism as predictors of antidepressant response: meta-analysis of three treatment cohorts. PLoS ONE.

[59] Lek M, Karczewski KJ, Minikel EV, Samocha KE, Banks E, Fennell T (2016). Analysis of protein-coding genetic variation in 60,706 humans. Nature.

