# Supplementary material for: Genetic contributions to alcohol use disorder treatment outcomes: a genome-wide pharmacogenomics study
Source: Neuropsychopharmacology. 2021 Jul 23;46(12):2132–9. doi: 10.1038/s41386-021-01097-0 (PMC8505452; doi:10.1038/s41386-021-01097-0)
Supplement: Supplementary file 1 — Supplemental Material [file 41386_2021_1097_MOESM1_ESM.docx]

Supplement to: Genetic Contributions to Alcohol Use Disorder Treatment Outcomes: A Genome-wide Pharmacogenomics Study

Contents:

Supplementary Methods: Samples

Supplementary Methods: Genotyping, QC and Imputation

Supplementary Methods: Gene-Level Associations and Gene-Set and Tissue Enrichment Analyses

Supplementary Results: Gene-level, Gene-Set, and Tissue Enrichment Analyses

Supplementary Table S1: Characteristics of the three contributing AUD treatment studies

Supplementary Table S2: Loci with suggestive evidence of association (p<5E-06) with one of the outcome measures when analyzing the full cohort (all treatments)

Supplementary Table S3: Loci with suggestive evidence of association (p<5E-06) with one of the outcome measures in analyses limited to patients treated with naltrexone

Supplementary Table S4: Loci with suggestive evidence of association (p<5E-06) with one of the outcome measures in analyses limited to patients treated with acamprosate

Supplementary Figure S1: QQ plots for time until relapse and time until heavy relapse in the analysis of data from all patients

Supplementary Figure S2: Regional association plot for the BRE locus in the full cohort analysis of time until heavy relapse.

Supplementary Figure S3: Manhattan and QQ plots for time until relapse and time until heavy relapse in naltrexone-treated patients

Supplementary Figure S4: Manhattan and QQ plots for time until relapse and time until heavy relapse in acamprosate-treated patients

Supplementary Figure S5: Forest Plots showing the individual study and meta-analysis results for the top SNPs in the full cohort and drug-stratified analyses

Supplementary Figure S6: MAGMA Tissue Expression Analysis of THR in the naltrexone-treated patients.

Supplementary Figure S7: Prediction of time until relapse (TR) and time until heavy relapse (THR) using polygenic risk scores (PRSs) for alcohol related traits.

**Supplementary Methods:**

*Samples*

This study used a new genomic dataset derived from three previously completed studies of acamprosate and/or naltrexone treatment of AUD: the COMBINE, PREDICT, and CITA studies [1-3]. The key characteristics of these three studies are summarized in Supplementary Table S1. The COMBINE study was a randomized placebo-controlled trial with 1383 alcohol dependent participants, comparing outcomes after 16 weeks of naltrexone or acamprosate, or both, with or without a combined behavioral intervention [1]. DNA for genetic studies was obtained from a subset of COMBINE study participants. PREDICT was a double-blind randomized trial comparing outcomes after three months of acamprosate, naltrexone, or placebo treatment in 426 AUD patients [3]. The CITA study was a single-arm pharmacogenomics study designed to investigate associations of acamprosate treatment outcomes with genetic polymorphisms in genes and pathways previously implicated in acamprosate response (N=443) [2]. All subjects included in our analyses provided consent allowing use of their clinical data and DNA for genetic studies of AUD and response to its treatment, and this study was approved by the Mayo Clinic Institutional Review Board.

*Genotyping, Quality Control (QC), and Imputation*

Male participants from the PREDICT study were previously genotyped using Illumina® HumanHap550v3 (Illumina, San Diego, CA, USA), Illumina® human610 (Illumina, San Diego, CA, USA), or Illumina® Human 660quad genotyping chips (Illumina, San Diego, CA, USA) as part of a GWAS of alcohol dependence [4]. Genetic data for SNPs present on all three platforms underwent QC, as previously described [4, 5]. Data for N=266 subjects and~464k SNPs passed QC and were available for our pharmacogenomics analyses.

CITA samples were first genotyped using Illumina® HumanCore (Illumina, San Diego, CA, USA) (N=433) arrays at the Medical Genome Facility at Mayo Clinic, and subsequently were re-genotyped using the denser Infinium® OmniExpressExome-8 BeadChips (Illumina, San Diego, CA, USA) (N=437, including N=400 previously genotyped samples) at National Institute on Alcohol Abuse and Alcoholism (NIAAA) Laboratory of Neurogenetics. Data from the two arrays were quality-controlled, combined and checked for concordance, and additional QC was performed on the combined dataset. Samples were excluded from analysis if they had a low call rate, extreme heterozygosity, or disagreement between reported sex and genetically determined sex. Sample relatedness was checked by pairwise identical-by-descent (IBD) estimation and one subject was removed from each pair with proportion IBD (i.e. PI_HAT) >.2. SNPs were excluded from analysis if they had a call rate <99% or deviated significantly from Hardy Weinberg Equilibrium (p<1E-06). Genetic data from 436 subjects passed QC, all with call rates >.995 and >70% European ancestry based on STRUCTURE [6] analysis with the 1000 genomes data as a reference sample were retained for analysis.

Of the 1383 subjects enrolled in the COMBINE study, DNA from 758 subjects was genotyped using Infinium® OmniExpressExome-8 BeadChips (Illumina, San Diego, CA, USA) at NIAAA Laboratory of Neurogenetics, and Standard QC was performed similarly to the CITA sample (excluding SNPs and samples with call rates <.95, SNPs with HWE p<1E-06, samples with discrepancies between reported sex and genetically determined sex, and one sample from each pair with IBD PI_HAT>.2). Additional QC filters were then applied, including genotype concordance checks with a prior candidate gene panel to identify potential sample mismatches. After QC, 505 subjects with >75% European ancestry based on STRUCTURE [6] analysis with the 1000 genomes data as a reference sample were retained for analysis.

Analyses were restricted to subjects of European ancestry. Imputation was performed separately for each study (436 CITA, 266 PREDICT and 505 COMBINE subjects of European ancestry) using the Michigan Imputation Server with the HRC reference panel (version HRC.r1-1.GRCh37.wgs.mac5.sites). For each dataset, variants with imputation dosage R^2^ <.5 and MAF <0.01 were excluded from analysis.

*Gene Level Associations and Gene-Set and Tissue Enrichment Analyses*

Gene-level tests were performed using Multi-marker Analysis of GenoMic Annotation (MAGMA) [7] as implemented in functional mapping and annotation of genetic associations (FUMA) [8], which uses Brown’s method to combine SNP p-values across a gene, while accounting for linkage disequilibrium (LD) between SNPs (i.e. r^2^). SNPs were assigned to genes based on being within 10kb of the gene and all SNPs in a gene region were included regardless of their functional class. Competitive gene-set analyses accounting for potential confounders such as gene size and LD were also performed using MAGMA in FUMA. MAGMA implementation in FUMA was further used to perform tissue enrichment analyses that test whether association results are enriched for genes expressed in particular tissue types based on Genotype-Tissue Expression (GTEx) data [9].

**Supplementary Results:**

*Gene-level, Gene-Set, and Tissue Enrichment Analyses*

There were no significant gene-level associations based on MAGMA analyses (see Methods for details) of the GWAS results presented above, although the *BRE* gene association with THR in the full sample approached significance (p=4.0E-6, Bonferroni-corrected p=0.079). In MAGMA gene-set association analyses of THR in naltrexone-treated patients, the strongest association was with a curated gene set named “boyault_liver_cancer_subclass_g56_up” representing up-regulated genes in hepatocellular carcinoma subclass G56, defined by unsupervised clustering; the 11 genes in this gene set showed enrichment for association with THR in the naltrexone-treated patients (p=1.0E-06, Bonferroni corrected p=0.016; most strongly associated genes in this gene set were *DPP4*, *SMYD2*, and *TBX3*). Also, in the analyses of naltrexone treatment outcomes, the top tissues based on expression of genes enriched for association signals (at p<0.05) were brain tissues, including the hippocampus, putamen, basal ganglia, cortex, and amygdala (Supplementary Figure S6), but these enrichment results were not statistically significant after multiple testing correction.

**Supplementary Table S1:** Characteristics of the three contributing AUD treatment studies.

|  | **COMBINE** | **PREDICT** | **CITA** |
| --- | --- | --- | --- |
| Study design | Double-blind randomized controlled trial  (Non-medication interventions were not blinded to participants) | Double-blind randomized controlled trial | Open-label uncontrolled trial  (Designed to identify genetic markers associated with treatment response) |
| Study treatment | Nine arms:   1. Medical Management + Medication:    1. Naltrexone & acamprosate    2. Naltrexone    3. Acamprosate    4. Placebo 2. Medical Management + Medication + CBI:    1. Naltrexone & acamprosate    2. Naltrexone    3. Acamprosate    4. Placebo 3. CBI only (no pills)   *Dose: Naltrexone (2×50 mg/d)  *Dose: Acamprosate (3×2tablets×500mg) | Three arms:   1. Naltrexone (1×50 mg/d) 2. Acamprosate (3×2tablets×333mg) 3. Placebo   *With biweekly Medical Management, i.e., manualized supportive therapy (until week 24)  *If relapsed, subjects were offered an inpatient intervention followed by re-randomization to either Medical Management or Cognitive Behavioral Intervention | One arm:   1. Acamprosate (3×2tablets×333mg)   *Attendance and involvement of Alcoholics Anonymous were encouraged and self-monitored |
| Treatment received at recruitment | Various (7.7% received inpatient detoxification service within 30 days prior to randomization) | Inpatients detoxification program | Inpatients detoxification program or outpatient program  *Comorbid depression and/or anxiety disorders were treated with antidepressants when necessary |
| Drug treatment trial period | 16 weeks | 12 weeks | 24 weeks |
| Medication compliance check | Self-report based on timeline-follow back and pill count | Self-report and pill count | Self-report and pill count |
| Follow-up schedules | Medical Management: weeks 0, 1, 2, 4, 6, 8, 10, 12, and 16  CBI: up to 20 sessions | Biweekly for Medical Management, then in-person visits: months 9, 12, 15, and 18 | In-person visits: months 1, 3, and 6  Phone calls: months 2, 4, and 5 |

| **(Cont’d)** | **COMBINE** | **PREDICT** | **CITA** |
| --- | --- | --- | --- |
| Sites of recruitment | U.S.A.: 11 academic sites | Germany: 5 Academic/University health centers and 2 psychiatric state hospitals | U.S.A.: Academic medical center (Mayo Clinic at Rochester, and 3 Mayo Health System sites) |
| Inclusion criteria | - Males or females age ≥21 years - Current DSM-IV diagnosis of alcohol dependence - Minimum of 14 drinks (females) or 21 drinks (males) on average per week over a consecutive 30-day period AND ≥2 days of heavy drinking (4 drinks for females, 5 drinks for males) within the 90 days prior to initiation of abstinence - At least 72 hours of abstinence and no significant withdrawal symptoms (CIWA<8) prior to randomization - Maximum of 21 days of abstinence prior to randomization - ≤21 consecutive days of planned absence during the 6-month active treatment period - A witnessed declaration of informed consent signed | - Males or females age >18 but < 65 years - Current DSM-IV/ICD-10 diagnosis of alcohol dependence - Minimum of 14 drinks (females) or 21 drinks (males) on average per week over a consecutive 30-day period AND ≥2 days of heavy drinking (4 drinks for females, 5 drinks for males) within the 90 days prior to initiation of abstinence - At least 72 hours of abstinence and no significant withdrawal symptoms (CIWA<8) prior to randomization - At least 2 weeks of inpatient detoxification - Maximum of 28 days of abstinence prior to randomization - Agree not to seek additional psychotherapy during the first 6 months of study (except mutual help groups) - A witnessed declaration of informed consent signed | - Male or females age 18-80 years - Current primary diagnosis of alcohol dependence based on DSM-IV-TR criteria - Last drink at least 5 days but not more than 6 months prior to enrollment - Enrollment in the IRB approved protocol “Developing a DNA Repository for Genomic Studies of Addiction” |
| Exclusion criteria | - Concurrent DSM-IV criteria for depression, bipolar disorder, schizophrenia, bulimia/anorexia, dementia, or a psychological disorder for whom medication is indicated (but not other Axis I disorders that are unmedicated) - Intend to engage with concurrent psychiatric treatment for alcohol-related problems - Require concomitant therapy with any medications that pose safety issues - Medical history of medical disorders that would increase potential risk of study treatment or interfere with study participation - Had more than 7 days of inpatient treatment for substance use disorder in the 30 days prior to randomization - History of other psychoactive substance abuse or dependence (other than nicotine, cannabis, and habitual caffeine use) by DSM-IV criteria in the last 90 days (6 months for opiate abuse) or by urine drug screen - Abnormal AST or ALT (>3 times of normal level) or elevated bilirubin - Pregnancy or nursing - Women of childbearing age not on an effective contraceptive method - Sensitivity to the study medications - Unstable medical conditions (e.g., serum liver enzyme levels >3 times the upper limit of normal) - No contact person who could provide the whereabouts of the participant and without a fixed address or unavailable by phone or pager at the time of randomization - Illiteracy or unable to read English | - Concurrent DSM-IV criteria for depression, bipolar disorder, schizophrenia, bulimia/anorexia, dementia, anxiety disorders - Required psychotherapy - On antidepressants, mood stabilizers, antiepileptics or other medications - Use of any psychoactive drugs as evident by urine test in the last 30 days - Lifetime diagnosis of psychoactive substance dependence (except nicotine and coffee) - Medical conditions that would increase the potential risk of the study treatment or interfere with the study participation - Abnormal AST or ALT levels (>5 times of normal level) - Pregnancy or nursing - Women of childbearing age not on an effective contraceptive method - Sensitivity to the study medication - Illiteracy or unable to read German | - Any unstable active medical or additional psychiatric conditions - Active suicidal ideation - History of hypersensitivity or allergic reaction to acamprosate - Currently taking disulfiram - Currently being, or within the last 3 weeks having been treated with acamprosate - Abnormal ALT or AST levels (>3 times of normal level) - Diagnosis of primary biliary cirrhosis, chronic active hepatitis, and drug-induced hepatic insufficiency, as noted in the medical record - Moderate to severe renal impairment (creatinine level >1.5 mg/dL) - Pregnancy, plan of pregnancy in the next year, or nursing - Unable to provide informed consent - Unable to speak English |

CBI=Combined Behavioral Intervention; DSM-IV=Diagnostic and Statistical Manual of Mental Disorders, Fourth Edition; CIWA=Clinical Institute Withdrawal Assessment for Alcohol; ICD=International classification of diseases; TR=time until relapse; DNA=Deoxyribonucleic acid; ALT=alanine transaminase; AST=Aspartate aminotransferase

**Supplementary Table S2:** Loci* with suggestive evidence of association (p<5E-06) with one of the outcome measures when analyzing the full cohort (all treatments)

| Outcome | rsID | chr:position | MA | CA | MAF | HR | Dir | P.value | Gene Annotation |
| --- | --- | --- | --- | --- | --- | --- | --- | --- | --- |
| Time Until Relapse | rs1078110 | 1:41252782 | C | G | 0.304 | 0.67 | --- | 6.2E-07 | intronic (KCNQ4[0]) |
|  | rs3097240 | 5:38605357 | G | A | 0.403 | 0.71 | --- | 9.9E-07 | ncRNA_intronic (LIFR-AS1[0]) |
|  | rs584789 | 5:118087436 | C | A | 0.303 | 1.37 | +++ | 2.0E-06 | intergenic (RP11-2N5.1[124]; RNU7-34P[6]) |
|  | rs77583603 | 2:186954132 | G | A | 0.089 | 1.69 | +++ | 2.0E-06 | intergenic (AC097500.2[6]; AC104058.1[74]) |
|  | rs433374 | 11:36994253 | C | A | 0.172 | 1.48 | +++ | 3.3E-06 | intergenic (CTD-2119L1.1[275]; SNORA31[729]) |
|  | rs79484822 | 4:70932193 | C | T | 0.095 | 1.60 | +++ | 4.5E-06 | upstream (CSN1S2AP[0]) |
|  | rs113018018 | 4:4887897 | A | G | 0.149 | 1.50 | +++ | 5.0E-06 | intergenic (MSX1[22]; LDHAP1[8]) |
|  |  |  |  |  |  |  |  |  |  |
| Time Until Heavy Relapse | **rs56951679** | **2:28483718** | **C** | **T** | **0.174** | **1.53** | **+++** | **1.6E-08** | **intronic (BRE[0])** |
|  | rs3019626 | 11:61808272 | t | c | 0.216 | 0.66 | --- | 1.7E-07 | intergenic (RP11-810P12.1[28]; RP11-810P12.6[32]) |
|  | rs2097759 | 17:36079453 | C | A | 0.153 | 0.63 | --- | 2.9E-06 | intronic (HNF1B[0]) |
|  | rs2015157 | 19:9346762 | G | T | 0.074 | 1.67 | +++ | 3.2E-06 | ncRNA_exonic (OR7D1P[0]) |
|  | rs11608708 | 12:132123936 | G | A | 0.188 | 1.46 | +++ | 3.5E-06 | intergenic (RP11-495K9.9[16]; RP11-495K9.7[8]) |

| MA=minor allele; CA=common allele; MAF=minor allele frequency; HR=hazard ratio reported in terms of MA. HR>1 indicates a higher risk of relapse/heavy-relapse associated with an additional copy of the MA, whereas HR<1 indicates a lower risk. Dir=Direction of effect (HR>1 or HR<1) in the three contributing studies (ordered as: COMBINE/CITA/PREDICT) |
| --- |
| *results were clumped using r^2^ of .1 within 250kb regions |

**Supplementary Table S3:** Loci* with suggestive evidence of association (p<5E-06) with one of the outcome measures in analyses limited to patients treated with naltrexone

| Outcome | rsID | chr:position | MA | CA | MAF | HR | Dir | P.value | Gene Annotation |
| --- | --- | --- | --- | --- | --- | --- | --- | --- | --- |
| Time Until Relapse | rs62533259 | 9:9535958 | C | T | 0.140 | 2.20 | ++ | 7.7E-08 | intronic (PTPRD[0]) |
|  | rs11588477 | 1:228512036 | G | T | 0.223 | 1.87 | ++ | 8.6E-08 | intronic (OBSCN[0]) |
|  | rs7325923 | 13:24078596 | T | A | 0.109 | 2.14 | ++ | 2.1E-07 | intergenic (LINC00352[2]; TNFRSF19[66]) |
|  | rs1362196 | 7:34413651 | G | A | 0.058 | 2.75 | ++ | 9.3E-07 | ncRNA_intronic (NPSR1-AS1[0]) |
|  | rs117970302 | 15:88518599 | T | C | 0.094 | 2.13 | ++ | 1.2E-06 | intronic (NTRK3[0]) |
|  | rs11770131 | 7:23872506 | A | T | 0.070 | 2.42 | ++ | 1.3E-06 | downstream (STK31[0]) |
|  | rs114469564 | 21:43404351 | G | T | 0.050 | 2.57 | ++ | 1.7E-06 | intergenic (C2CD2[30]; ZBTB21[3]) |
|  | rs2908000 | 7:9898826 | C | T | 0.334 | 0.57 | -- | 1.8E-06 | intergenic (GS1-69O6.1[8]; AC006373.1[102]) |
|  | rs7828702 | 8:3003780 | G | A | 0.101 | 2.06 | ++ | 1.8E-06 | intronic (CSMD1[0]) |
|  | rs72765992 | 5:58579309 | C | G | 0.081 | 2.21 | ++ | 2.1E-06 | intronic (PDE4D[0]) |
|  | rs6076964 | 20:6210743 | T | C | 0.070 | 2.75 | ++ | 2.2E-06 | intergenic (AL109618.1[1]; CASC20[217]) |
|  | rs80081786 | 1:177797988 | C | T | 0.156 | 1.94 | ++ | 2.6E-06 | intergenic (RP11-63B19.1[119]; RP4-798P15.3[100]) |
|  | rs7141455 | 14:52227835 | T | C | 0.081 | 2.48 | ++ | 3.1E-06 | ncRNA_exonic (RP11-280K24.1[0]) |
|  | rs7187668 | 16:87077722 | T | C | 0.111 | 0.41 | -- | 3.5E-06 | intergenic (RP11-107C10.1[138]; RP11-134D3.1[14]) |
|  | rs12323856 | 14:105293185 | G | T | 0.051 | 6.54 | +? | 3.8E-06 | intergenic (LINC00638[3]; RPS26P49[4]) |
|  | rs11010438 | 10:36386254 | T | G | 0.123 | 1.84 | ++ | 4.8E-06 | intergenic (RP11-810B23.1[8]; MTND5P17[336]) |
| Time Until Heavy Relapse | **rs12749274** | **1:56883491** | **a** | **g** | **0.079** | **2.90** | **++** | **3.9E-08** | **intergenic (RP4-710M16.2[2]; PPAP2B[77])** |
|  | rs62533259 | 9:9535958 | C | T | 0.138 | 2.36 | ++ | 6.1E-08 | intronic (PTPRD[0]) |
|  | rs7141455 | 14:52227835 | t | c | 0.081 | 2.70 | ++ | 4.8E-07 | ncRNA_exonic (RP11-280K24.1[0]) |
|  | rs71569385 | 6:37575654 | T | C | 0.050 | 3.14 | ++ | 7.6E-07 | intergenic (MIR4462[52]; MDGA1[25]) |
|  | rs78950771 | 5:154582312 | G | A | 0.070 | 2.73 | ++ | 1.9E-06 | intergenic (CTD-2311A18.1[90]; CTC-447K7.1[195]) |
|  | rs148035692 | 16:5143629 | a | g | 0.069 | 4.57 | ?+ | 2.0E-06 | intronic (FAM86A[0]) |
|  | rs57326784 | 1:209836879 | T | C | 0.065 | 3.70 | -+ | 2.0E-06 | ncRNA_intronic (RP1-28O10.1[0]) |
|  | rs11588477 | 1:228512036 | G | T | 0.225 | 1.79 | ++ | 2.0E-06 | intronic (OBSCN[0]) |
|  | rs34428249 | 10:88763843 | G | A | 0.075 | 2.46 | ++ | 2.8E-06 | ncRNA_intronic (AGAP11[0]; RP11-96C23.11[0]; RP11-96C23.5[0]; RP11-96C23.14[0]) |
|  | rs10788250 | 10:123901712 | t | c | 0.080 | 2.30 | ++ | 4.7E-06 | intronic (TACC2[0]) |

| MA=minor allele; CA=common allele; MAF=minor allele frequency; HR=hazard ratio reported in terms of MA. HR>1 indicates a higher risk of relapse/heavy-relapse associated with an additional copy of the MA, whereas HR<1 indicates a lower risk. Dir=Direction of effect (HR>1 or HR<1) in the three contributing studies (ordered as: COMBINE/PREDICT) |
| --- |
| *results were clumped using r^2^ of .1 within 250kb regions |

**Supplementary Table S4:** Loci* with suggestive evidence of association (p<5E-06) with one of the outcome measures in analyses limited to patients treated with acamprosate

| Outcome | rsID | chr:position | MA | CA | MAF | HR | Dir | P.value | Gene Annotation |  |
| --- | --- | --- | --- | --- | --- | --- | --- | --- | --- | --- |
| Time Until Relapse | **rs77583603** | **2:186954132** | **G** | **A** | **0.093** | **2.38** | **+++** | **3.1E-09** | **intergenic (AC097500.2[6]; AC104058.1[74])** |  |
|  | rs78024421 | 13:58883475 | C | T | 0.059 | 2.52 | +++ | 8.8E-07 | intergenic (LINC00374[76]; RNY4P29[218]) |  |
|  | rs4582229 | 5:60848745 | T | C | 0.211 | 0.56 | --- | 1.1E-06 | intergenic (ZSWIM6[7]; AC008836.1[4]) |  |
|  | rs7233031 | 18:5480461 | G | A | 0.271 | 1.57 | +++ | 1.9E-06 | ncRNA_intronic (RP11-286N3.1[0]) |  |
|  | rs2435356 | 10:43583150 | A | G | 0.250 | 1.56 | +++ | 2.9E-06 | intronic (RET[0]) |  |
|  | rs10965428 | 9:22718481 | C | A | 0.086 | 1.90 | +++ | 3.0E-06 | ncRNA_intronic (RP11-399D6.2[0]) |  |
|  | rs11601116 | 11:36986344 | T | C | 0.187 | 1.63 | +++ | 3.0E-06 | intergenic (CTD-2119L1.1[267]; SNORA31[737]) |  |
|  | rs72684759 | 14:21889563 | T | C | 0.129 | 2.14 | +?+ | 3.2E-06 | intronic (CHD8[0]) |  |
|  | rs27565 | 5:59837591 | C | T | 0.468 | 0.67 | --- | 3.5E-06 | ncRNA_intronic (PART1[0]) |  |
|  | rs7249601 | 19:57546158 | C | T | 0.066 | 2.03 | +++ | 4.0E-06 | intergenic (CTC-258N23.3[113]; RPL7AP69[31]) |  |
|  | rs213041 | 1:21657769 | T | C | 0.087 | 1.87 | +++ | 4.2E-06 | intronic (ECE1[0]) |  |
|  | rs702131 | 9:12526192 | G | A | 0.279 | 1.54 | +++ | 4.2E-06 | intergenic (RNU2-47P[226]; TYRP1[159]) |  |
|  | rs35734605 | 13:50149649 | T | C | 0.094 | 2.43 | +?+ | 4.5E-06 | intronic (RCBTB1[0]) |  |
|  | rs9924363 | 16:19686316 | C | A | 0.485 | 0.66 | --- | 4.9E-06 | intronic (C16orf62[0]) |  |
| Time Until Heavy Relapse | rs34797278 | 17:14311624 | G | A | 0.079 | 2.14 | +++ | 5.4E-08 | ncRNA_intronic (AC022816.2[0]) |  |
|  | rs79234780 | 10:82585091 | A | G | 0.052 | 2.24 | +++ | 3.5E-07 | intergenic (FARSBP1[47]; WARS2P1[115]) |  |
|  | rs113943471 | 6:88519981 | t | c | 0.077 | 2.85 | +?+ | 4.8E-07 | intergenic (snoU13[89]; Y_RNA[16]) |  |
|  | rs6087091 | 20:1050896 | t | c | 0.234 | 1.57 | +++ | 1.4E-06 | intergenic (RSPO4[68]; PSMF1[43]) |  |
|  | rs12702045 | 7:43507689 | a | c | 0.222 | 1.66 | +++ | 1.4E-06 | intronic (HECW1[0]) |  |
|  | rs10843660 | 12:30368457 | t | c | 0.421 | 0.65 | --- | 1.6E-06 | ncRNA_intronic (RP11-776A13.1[0]) |  |
|  | rs6865889 | 5:123683057 | t | c | 0.084 | 1.92 | +++ | 1.6E-06 | ncRNA_intronic (LINC01170[0]) |  |
|  | rs17160835 | 7:138948598 | G | A | 0.197 | 1.59 | +++ | 2.1E-06 | intronic (UBN2[0]) |  |
|  | rs400781 | 19:53138219 | A | G | 0.055 | 2.24 | +++ | 2.2E-06 | intronic (ZNF83[0]) |  |
|  | rs13298120 | 9:36640907 | t | c | 0.165 | 1.68 | +++ | 2.6E-06 | intronic (MELK[0]) |  |
|  | rs288485 | 7:130450214 | G | A | 0.052 | 2.22 | +++ | 2.7E-06 | intergenic (KLF14[31]; RP11-36B6.1[25]) |  |
|  | rs75466853 | 13:59395212 | G | A | 0.065 | 2.09 | +++ | 3.1E-06 | intergenic (DNAJA1P1[69]; HMGN2P39[189]) |  |
|  | rs73650194 | 9:22749908 | T | A | 0.057 | 2.04 | +++ | 3.3E-06 | ncRNA_intronic (RP11-399D6.2[0]) |  |
|  | rs56010289 | 7:28468621 | G | C | 0.068 | 2.14 | +++ | 3.6E-06 | intronic (CREB5[0]) |  |
|  | rs2435356 | 10:43583150 | a | g | 0.246 | 1.55 | +++ | 3.9E-06 | intronic (RET[0]) |  |
|  | rs1335855 | 1:34648046 | C | T | 0.129 | 1.68 | +++ | 4.5E-06 | intronic (C1orf94[0]) |  |
| MA=minor allele; CA=common allele; MAF=minor allele frequency; HR=hazard ratio reported in terms of MA. HR>1 indicates a higher risk of relapse/heavy-relapse associated with an additional copy of the MA, whereas HR<1 indicates a lower risk. Dir=Direction of effect (HR>1 or HR<1) in the three contributing studies (ordered as: COMBINE/CITA/PREDICT). *results have been clumped using r^2^ of .1 within 250kb regions | | | | | | | | | | |

**Supplementary Figure S1:** QQ plots for meta-analyses of (A) Time until Relapse and (B) Time until Heavy Relapse, in the full cohorts (all treatments). Manhattan plots for the corresponding analyses are shown in Figure 1.

B

A


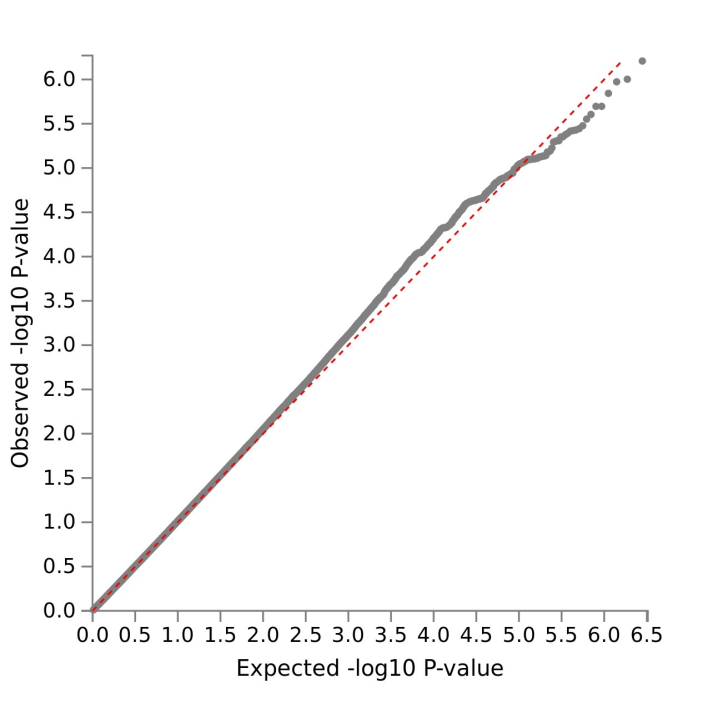

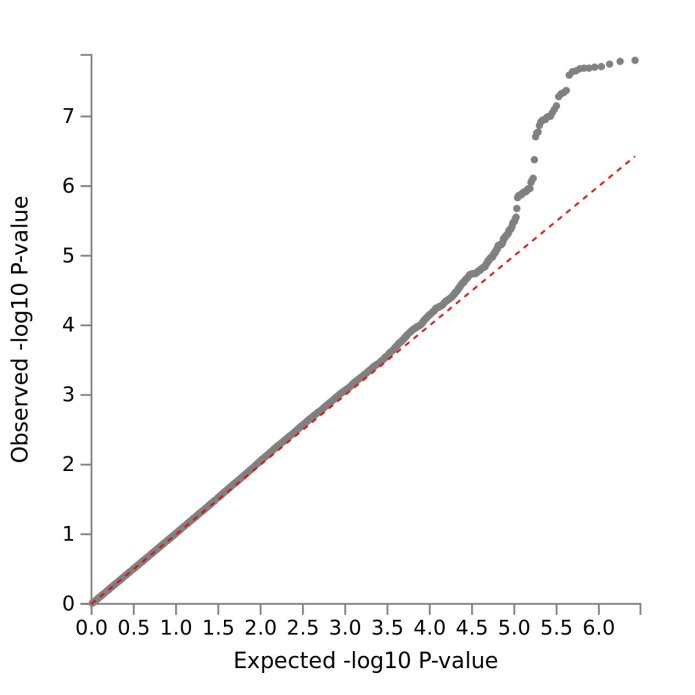


**Supplementary Figure S2**: Regional association plot for the BRE locus in the full cohort analysis of time until heavy relapse, generated using LocusZoom [10].

**Supplementary Figure S3:** Manhattan Plots for (A) TR and (B) THR in naltrexone-treated subjects. The corresponding QQ plots for TR and THR are shown in panels (C) and (D), respectively.

A


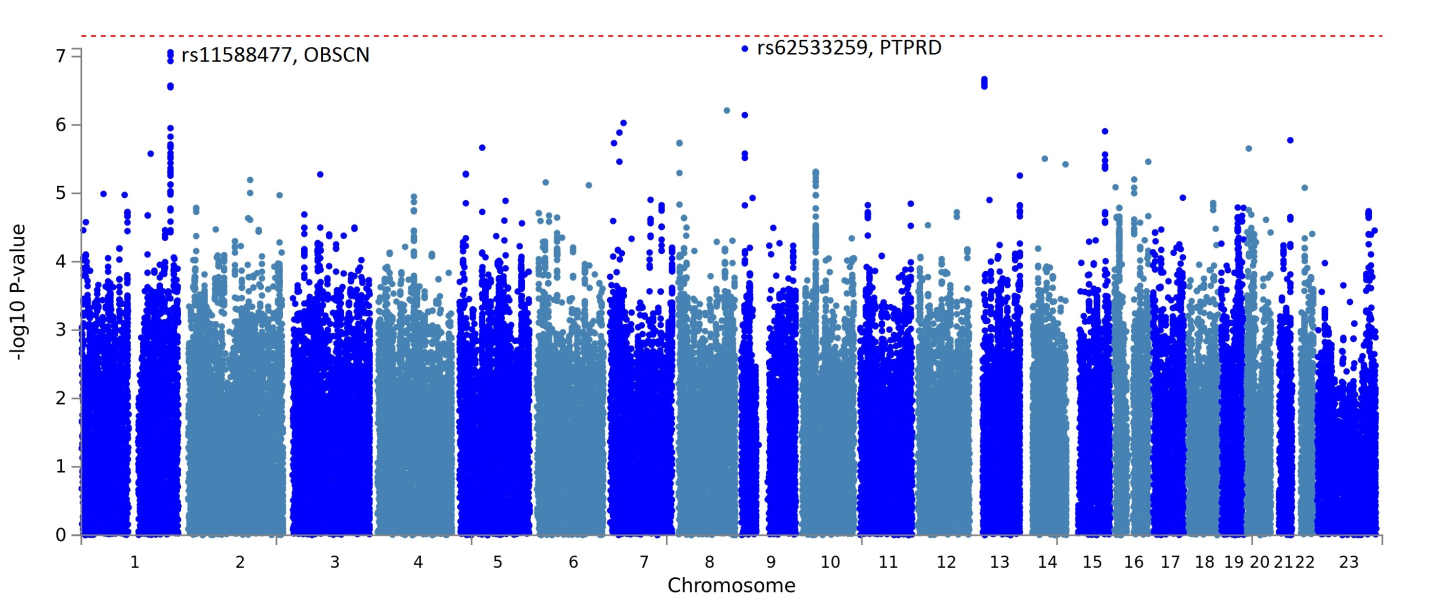


B


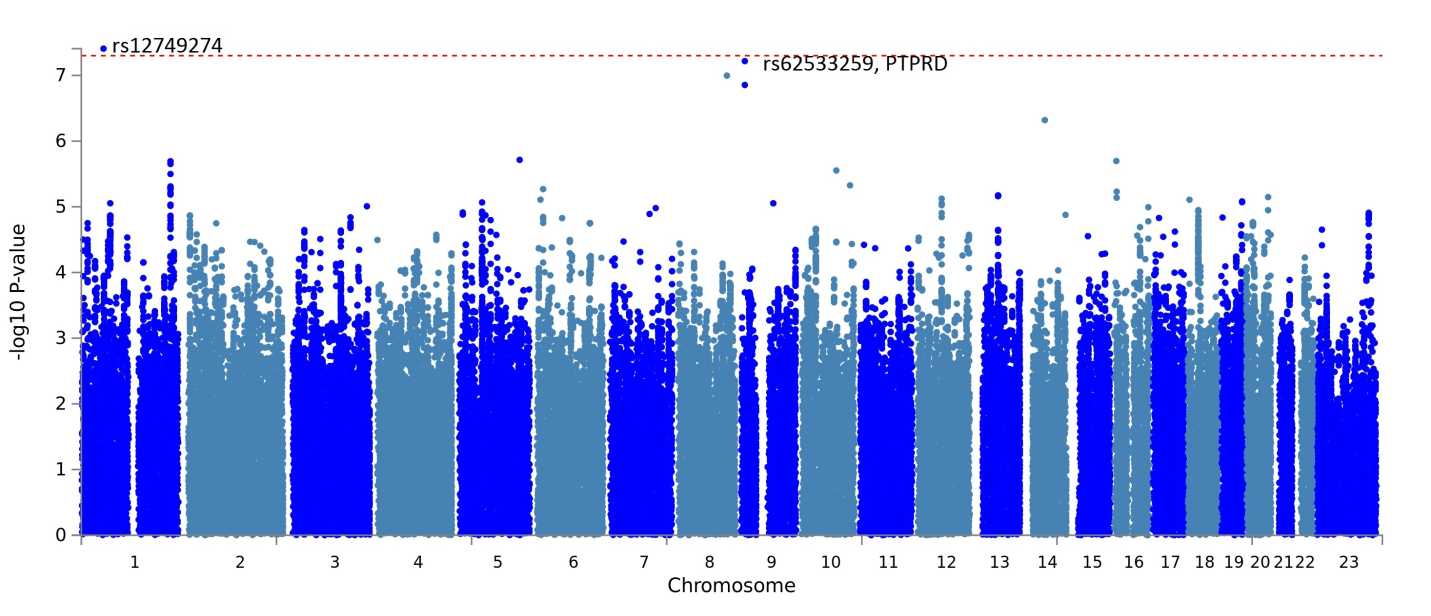


C D


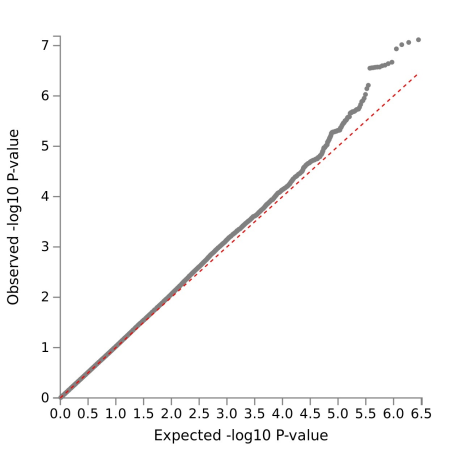

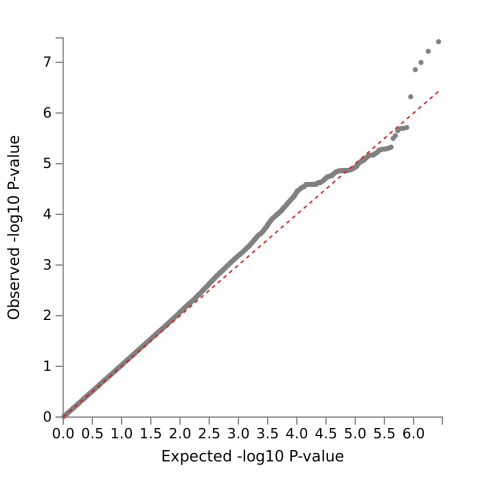


**Supplementary Figure S4:** Manhattan Plots for (A) TR and (B) THR in acamprosate-treated subjects. The corresponding QQ plots for TR and THR are shown in panels (C) and (D), respectively.

A


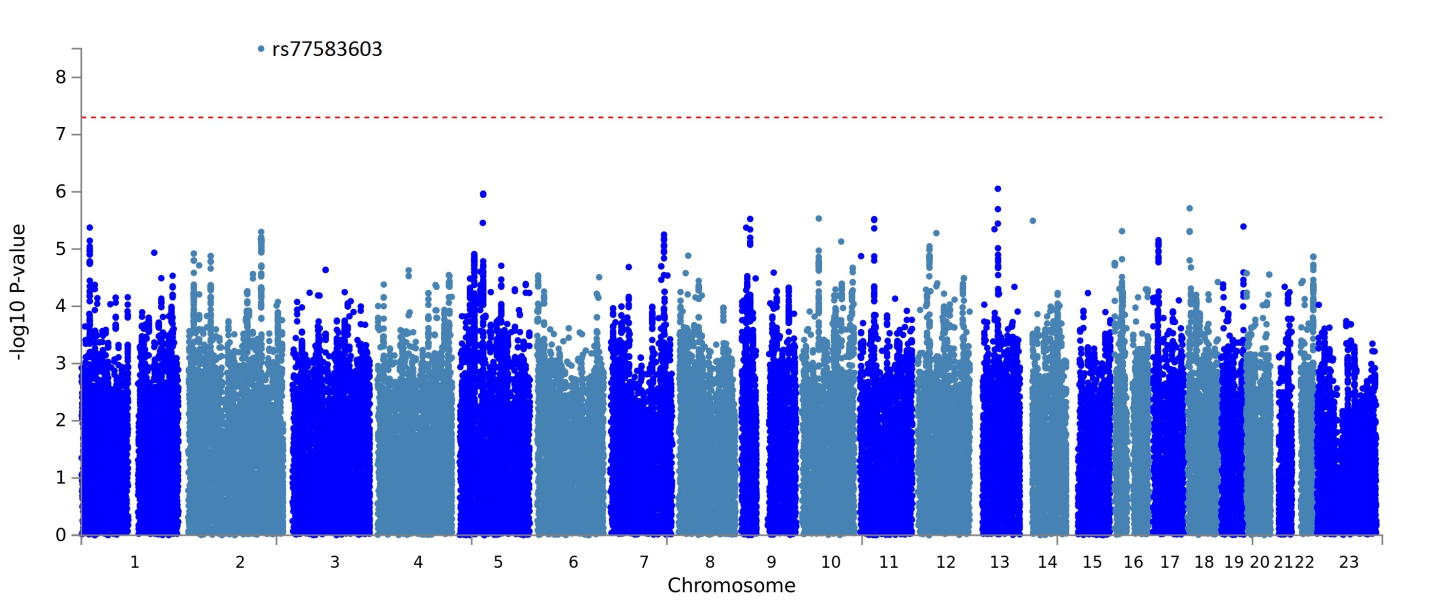


B

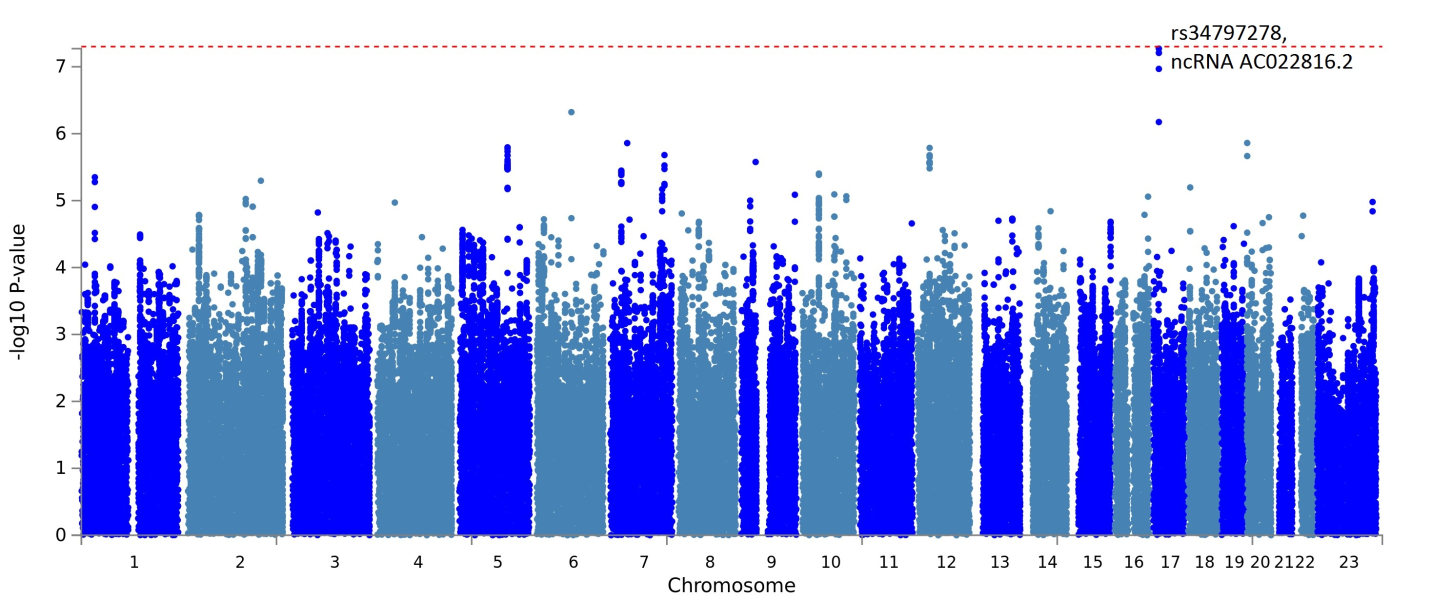


C D


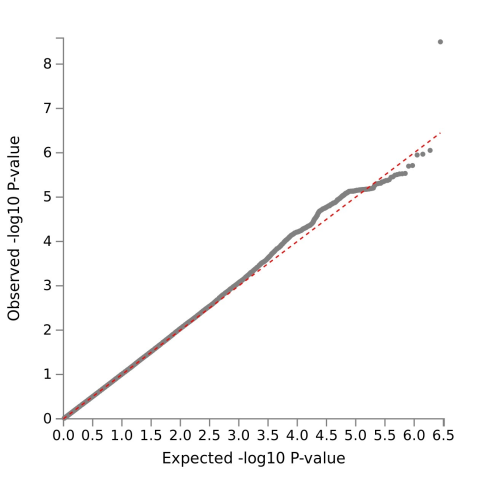

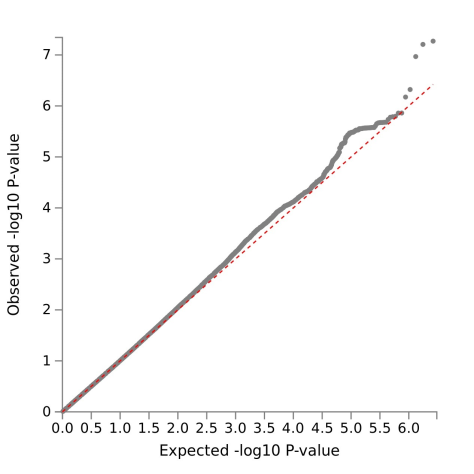


**Supplementary Figure S5**: Forest Plots showing the individual study and meta-analysis results (hazard ratios with 95% confidence intervals) for (A) the *BRE* SNP rs56951679 in the full cohort analysis of THR, (B) *PTPRD* SNP rs62533259 in the analysis of TR in naltrexone treated patients, (C) rs12749274 in the analysis of THR in naltrexone treated patients, (D) SNP rs77583603 in the TR analysis of acamprosate treated patients.

**B**

**A**


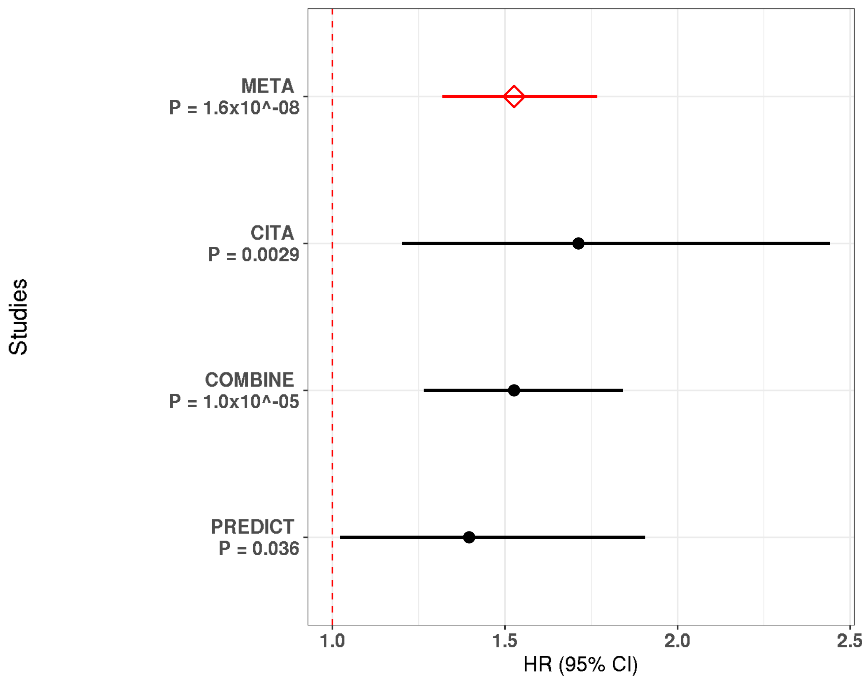

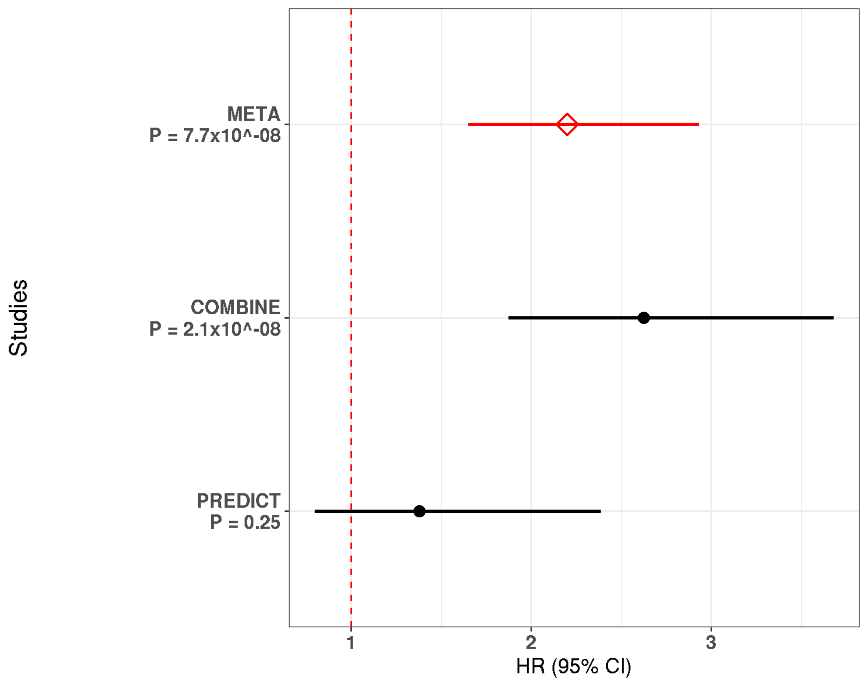


**C**

**D**


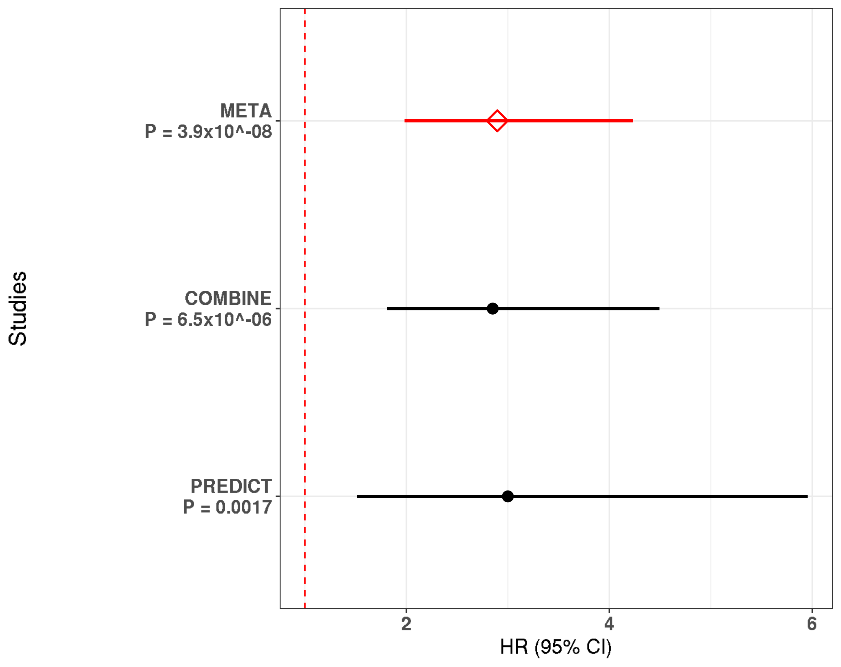

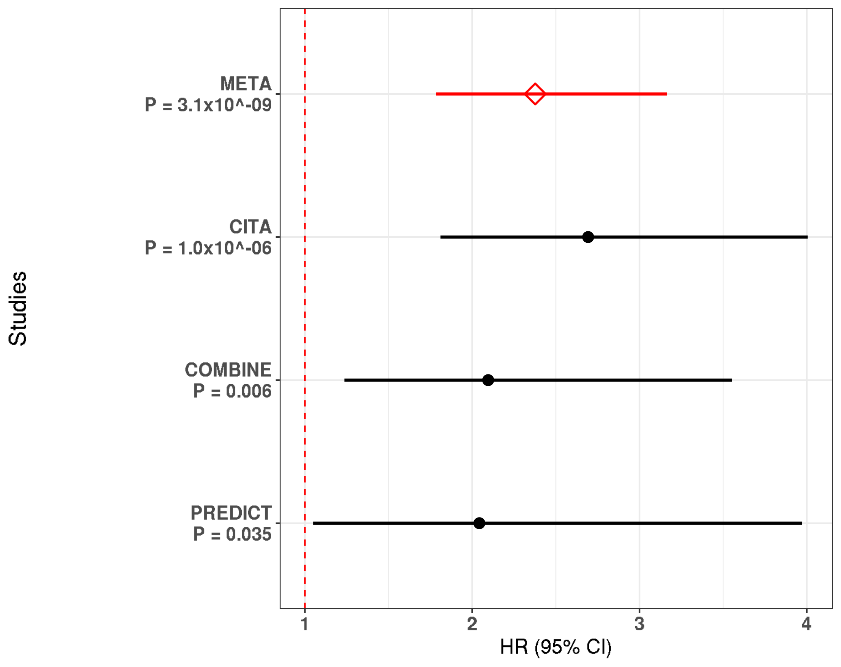


**Supplementary Figure S6:** MAGMA tissue enrichment analysis in the naltrexone-treated patients. This analysis tests for enrichment of differentially expressed gene sets in a given tissue compared to all other tissue types. The-log10(p-value) for the enrichment analysis of each analyzed tissue is shown on the y-axis.


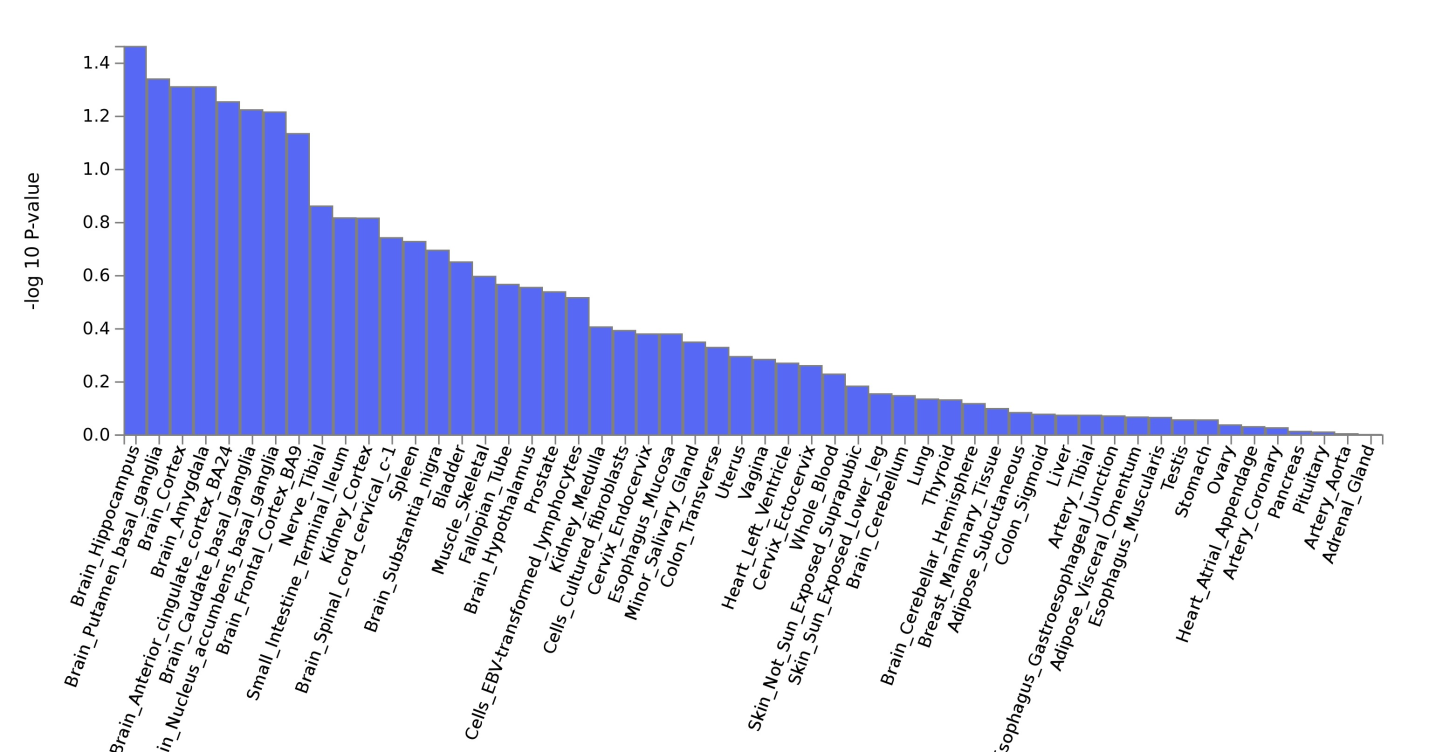


**Supplementary Figure S7**: Prediction of time until relapse (TR) and time until heavy relapse (THR) using polygenic risk scores (PRSs) for alcohol related traits. Details about each PRS including source of GWAS summary statistics, brief sample description, and effective sample size of the GWAS (Neff) are included in the labels on the left side of the figure. Forest plots show the 95% CI of the HR in each study and the combined meta-analysis for prediction of the outcome (TR or THR) using the PRS. P-values from the meta-analyses of association of PRS with TR and THR are shown in parentheses (to the left of the TR and THR Forest Plots).


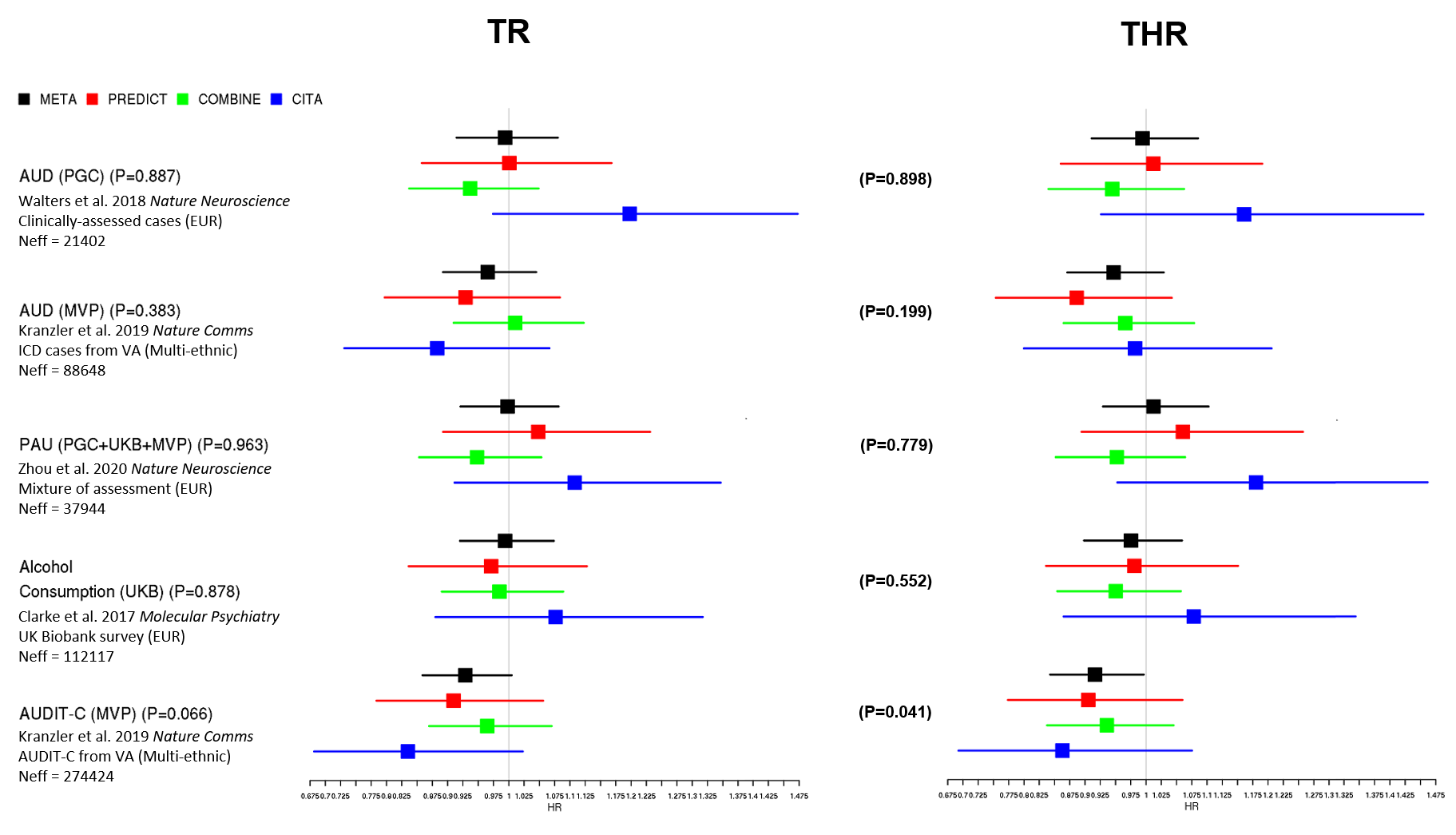


References

1. Anton, R.F., et al., *Combined pharmacotherapies and behavioral interventions for alcohol dependence: the COMBINE study: a randomized controlled trial.* JAMA : the journal of the American Medical Association, 2006. **295**(17): p. 2003-17.

2. Karpyak, V.M., et al., *Genetic markers associated with abstinence length in alcohol-dependent subjects treated with acamprosate.* Translational psychiatry, 2014. **4**: p. e453.

3. Mann, K., et al., *Searching for responders to acamprosate and naltrexone in alcoholism treatment: rationale and design of the PREDICT study.* Alcoholism, Clinical and Experimental Research, 2009. **33**(4): p. 674-83.

4. Treutlein, J., et al., *Genome-wide association study of alcohol dependence.* Arch Gen Psychiatry, 2009. **66**(7): p. 773-84.

5. Frank, J., et al., *Genome-wide significant association between alcohol dependence and a variant in the ADH gene cluster.* Addict Biol, 2012. **17**(1): p. 171-80.

6. Pritchard, J.K., M. Stephens, and P. Donnelly, *Inference of population structure using multilocus genotype data.* Genetics, 2000. **155**(2): p. 945-59.

7. de Leeuw, C.A., et al., *MAGMA: generalized gene-set analysis of GWAS data.* PLoS Comput Biol, 2015. **11**(4): p. e1004219.

8. Watanabe, K., et al., *Functional mapping and annotation of genetic associations with FUMA.* Nat Commun, 2017. **8**(1): p. 1826.

9. Consortium, G.T., *The Genotype-Tissue Expression (GTEx) project.* Nat Genet, 2013. **45**(6): p. 580-5.

10. Pruim, R.J., et al., *LocusZoom: regional visualization of genome-wide association scan results.* Bioinformatics, 2010. **26**(18): p. 2336-7.
